# Exploiting Protein-Protein Interaction Networks for Genome-Wide Disease-Gene Prioritization

**DOI:** 10.1371/journal.pone.0043557

**Published:** 2012-09-21

**Authors:** Emre Guney, Baldo Oliva

**Affiliations:** Structural Bioinformatics Group (GRIB), Universitat Pompeu Fabra, Barcelona Research Park of Biomedicine (PRBB), Barcelona, Catalonia, Spain; Aberystwyth University, United Kingdom

## Abstract

Complex genetic disorders often involve products of multiple genes acting cooperatively. Hence, the pathophenotype is the outcome of the perturbations in the underlying pathways, where gene products cooperate through various mechanisms such as protein-protein interactions. Pinpointing the decisive elements of such disease pathways is still challenging. Over the last years, computational approaches exploiting interaction network topology have been successfully applied to prioritize individual genes involved in diseases. Although linkage intervals provide a list of disease-gene candidates, recent genome-wide studies demonstrate that genes not associated with any known linkage interval may also contribute to the disease phenotype. Network based prioritization methods help highlighting such associations. Still, there is a need for robust methods that capture the interplay among disease-associated genes mediated by the topology of the network. Here, we propose a genome-wide network-based prioritization framework named GUILD. This framework implements four network-based disease-gene prioritization algorithms. We analyze the performance of these algorithms in dozens of disease phenotypes. The algorithms in GUILD are compared to state-of-the-art network topology based algorithms for prioritization of genes. As a proof of principle, we investigate top-ranking genes in Alzheimer's disease (AD), diabetes and AIDS using disease-gene associations from various sources. We show that GUILD is able to significantly highlight disease-gene associations that are not used *a priori*. Our findings suggest that GUILD helps to identify genes implicated in the pathology of human disorders independent of the loci associated with the disorders.

## Introduction

Genetic diversity is augmented by variations in genetic sequence, however not all the mutations are beneficial for the organism. Coupled with environmental factors these variations can disrupt the complex machinery of the cell and cause functional abnormalities. Over the past few decades, a substantial amount of effort has been exerted towards explaining sequential variations in human DNA and their consequences on human biology [1]. Linkage analysis [2], association studies and genome-wide association studies (GWAS) [3] have achieved considerable success in identifying causal loci of human disorders, albeit with limitations [1], [4].

Complex genetic disorders implicate several genes involved in various biological processes. Interactions of the proteins of these genes have helped extend our view of the genetic causes of common diseases [5]–[7]. Genes related to a particular disease phenotype (disease genes) have been demonstrated to be highly connected in the interaction network (e.g., in toxicity modulation [8] and cancer [9], [10]). Yet, rather than having random connections through the network, the interactions of proteins encoded by genes implicated in such phenotypes involve partners from similar disease phenotypes [11]–[13].

Linkage analysis typically associates certain chromosomal loci (linkage interval) with a particular disease phenotype. Such analysis produces a set of genes within the linkage interval. Recent studies have confirmed the usefulness of network-based approaches to prioritize such candidate disease genes based on their proximity to known disease genes (seeds) in the network. These studies can be distinguished by the way they define proximity between the gene products in the network of protein-protein interactions. Thus, proximity is defined by considering direct neighborhood [14]–[18], or by ranking with respect to shortest distance between disease genes [17], [19]–[21] or using methods based on random walk on the edges of the network [19], [22], [23]. Making use of the global topology of the network, random walk based methods have been shown to perform better than local approaches [19], [22], [24].

Two inherent properties of available data on protein-protein interactions (PPI) that affect the prioritization methods are incompleteness (false negatives) and noise (false positives). The bias towards highly connected known disease nodes in protein interaction networks has recently motivated statistical adjustment methods on the top of the association scores computed by prioritization algorithms where node scores are normalized using random networks [25]. Furthermore, taking network quality into consideration, several approaches incorporate gene expression and data on functional similarity in addition to physical PPIs [20], [26]–[31]. Gene prioritization is then based on the integrated functional network, redefining “gene-neighborhood” at the functional level.

Network-based approaches can also aid in identifying novel disease genes, even when the associated linkage intervals are not considered, for instance, to prioritize genes from GWAS [28], [32], [33]. In fact, using the whole genome to prioritize disease-gene variants is expected to produce more robust results in identifying modest-risk disease-gene variants than using high-risk alleles [34]. Nonetheless, existing prioritization methods substantially suffer from a lack of linkage interval information [24] and depend on the quality of the interaction network [25]. Thus, to identify genes implicated in diseases, stout methods that exploit interaction networks to capture the communication mechanism between genes involved in similar disease phenotypes are needed.

Available network-topology based prioritization methods treat all the paths in the network equally relevant for the pathology. We hypothesize that the communication between nodes of the network (proteins) can be captured by taking into account the “relevance” of the paths connecting disease-associated nodes. Here, we present GUILD (Genes Underlying Inheritance Linked Disorders), a network-based disease gene prioritization framework. GUILD proposes four topology-based ranking algorithms: NetShort, NetZcore, NetScore and NetCombo. Additionally, several other state-of-the-art algorithms that use global network topology have been included in GUILD: PageRank with priors [35] (as used in ToppNet [23]), Functional Flow [36], Random walk with restart [19] and Network propagation [22]. The framework uses known disease genes and interactions between the products of these genes. We show the effectiveness of the proposed prioritization methods developed under the GUILD framework for genome-wide prioritization. We also use several interaction data sets with different characteristics for various disease phenotypes to evaluate the classifier performance of these methods. As a proof of principle we use GUILD to pinpoint genes involved in the pathology of Alzheimer's disease (AD), diabetes and AIDS. GUILD is freely available for download at http://sbi.imim.es/GUILD.php.

## Results

### Comparison of GUILD with the state-of-the-art of network-topology based prioritization methods

We tested the prioritization algorithms in GUILD using three sources of gene-phenotypic association and the largest connected components of five different protein-protein interaction networks (see “Methods” for details and names of these sets). The area under ROC curve (AUC) was used to compare each ranking method (four novel methods NetScore, NetZcore, NetShort and NetCombo; and four existing state-of-the-art methods, Functional Flow, PageRank with priors, Random walk with restart and Network propagation). The AUCs for each method averaged over all disorders in different disease data sets (OMIM, Goh and Chen) and interaction data sets (Goh, Entrez, PPI, bPPI, weighted bPPI) are given in Table 1 (see Table S1 for the AUC values averaged over diseases on each interaction network separately). We also compared the ratio of seeds covered (sensitivity) among the top 1% predictions of each method (Table 1).

**Table 1:** 5-fold AUC (%) and sensitivity (%) at top 1% for each method averaged over all diseases within the data set and all interaction networks.*

Data Set	Metric	NetScore	NetZcore	NetShort	NetCombo	Func. Flow	PageRank	Random Walk	Network Prop.
OMIM	AUC	67.49	62.99	65.63	**72.09**	58.55	57.03	55.36	65.97
	Sens.	20.69	19.62	15.41	21.46	22.31	10.76	14.64	**23.24**
Goh	AUC	**67.32**	61.45	55.36	67.08	54.78	52.39	49.35	54.74
	Sens.	**11.61**	11.05	4.88	11.34	6.22	4.00	5.69	8.66
Chen	AUC	75.92	72.80	63.11	**78.41**	63.56	65.30	61.78	69.07
	Sens.	**18.89**	12.84	9.06	17.51	12.43	6.00	9.64	15.30

* Results are averaged over the interaction networks: Goh, Entrez, PPI, bPPI and weighted bPPI. OMIM, Goh and Chen denote the prioritization using data from OMIM, Goh et al. and Chen et al. respectively as explained in the text. The highest value in each row is highlighted.

In general, our methods produced more accurate predictions and better sensitivity in genome-wide prioritization than the up-to-date algorithms with which we compared. NetCombo, the consensus method combining NetScore, NetZcore and NetShort, proved to be an effective strategy of prioritization independent of the data set used. NetCombo produced significantly better predictions than Network Propagation, the best of the state-of-art tested approaches, on each data set (*P≤5.7e-6*, see Table S2 for associated p-values). Also the improvement of NetScore versus Network Propagation was significant in Goh and Chen data sets (*P≤8.2e-5*). Figure S1 compares the significant improvements in AUC.

We also tested alternative ways to combine prioritization methods. However, none of the combinations using other methods proved as effective as combining the three methods included in NetCombo. Details showing the average AUC and sensitivity among the top 1% high scoring genes of each disorder for each prioritization method using OMIM, Goh and Chen data sets on each interaction network can be found in Tables S3 and S4.

In order to avoid bias towards highly studied diseases we used equal number of gold standard positive and negative instances via grouping all the non-seed scores in *k* groups, where *k* is the number of seeds associated with the disease under evaluation (see “Methods”). Considering that the distribution of disease associated genes among all the genes is not known *a priori*, this assumption provided a fair testing set to compare different prediction methods than using all non-seeds as negatives or using only a random subsample of non-seeds. We also compared the prioritization methods when all non-seeds were assumed as negatives. The AUC values increased for all methods on all data sets (up to 10%). In all tests NetCombo and NetScore outperformed existing prioritization methods (see Table S5).

### Effect of the quality of the interaction network on discovering novel disease-genes

**Figure 1:**
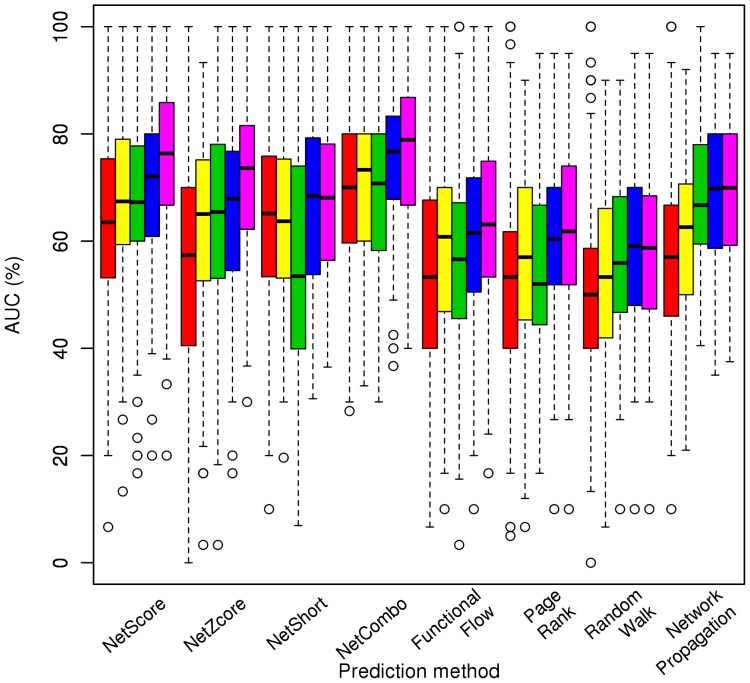
Prediction performance of GUILD approaches on each interaction network over all phenotypes of OMIM, Goh and Chen data sets. The distribution of AUCs for different phenotypes in each network is represented with a box-plot of different color. Color legend: red, Goh network; yellow, Entrez network; green, PPI network; blue, bPPI network; purple, weighted bPPI network.

The prediction performance of these methods depended on the topology of the network and the quality of the knowledge of protein-protein interactions in regards to size and reliability. We grouped the AUCs of all disorders by network type to test these dependencies (see “Methods” for network definitions). The distribution of AUCs for each interaction data set using OMIM, Goh and Chen data sets is given in Figure 1 (see Figure S2 for the distribution of sensitivity values with the top 1% predictions). Interestingly, most of the methods produced their best results with the weighted bPPI network, which used the scores from the STRING database [37] to weight the edges (see Table S1 for the average AUC). The improvement of the prediction performance using edge confidence values from STRING was significant for most methods (with the exception of NetShort and Random walk with restart algorithms, for which the performance improved but not significantly). These results justify the importance of network quality (i.e. using reliable binary interactions).

Furthermore, we hypothesized that removing interactions detected by pull down methods, such as Tandem Affinity Purification (TAP), would filter the noise produced by false binary interactions, consequently increasing the AUC and the sensitivity among top ranked predictions when the bPPI network was used instead of the PPI network (see Table S1).

Our results indicated that the network size was relevant too when binary interactions were used. The Goh network, which was smaller than the bPPI network, produced significantly lower AUC values for the majority of prioritization methods (all but NetShort). Thus, the use of the largest possible network with assessed binary interactions could improve the predictions. Based on the AUC values for each phenotype when the bPPI network is used, NetCombo, NetScore, NetZcore, and NetShort were significantly better than Functional Flow, PageRank with priors and Random walk with restart. NetCombo had an average AUC of 74.7% using the bPPI network on OMIM data set and this was the only method over 70% AUC (Table S1). However, when the weighted bPPI network was used to study the same data set, the AUCs of NetScore and NetZcore methods also surpassed this limit, with values around 74% and 72% respectively (NetCombo achieved 76.5% AUC in this case).

### Dependence on the connectivity between disease-associated genes

Next, we questioned whether the prediction methods depended on the connectivity between seeds using OMIM, Goh and Chen data sets. Table 2 shows the correlation between the average AUC of the prioritization methods and the graph features involving seeds of each disease phenotype in the bPPI network (number of seeds, number of neighboring seeds, and average shortest path length between seeds). A small inverse correlation was found between the average length of the shortest paths connecting seeds and the prediction capacity for all methods. This correlation was observed when using any of the interaction networks; therefore, it was independent of the underlying network. Average number of neighboring seeds also correlated with prediction performance, but less than the average length of the shortest paths connecting the seeds.

**Table 2:** Correlations between prediction performances of methods, measured as the average AUC over phenotypes, and seed connectivity values (associated p-values are included in parenthesis).

	Number of seeds	Average number of neighboring seeds	Average shortest path length between seeds
NetScore	−0.01 (0.86)	0.41 (1.1e-6)	−0.43 (2.1e-7)
NetZcore	−0.04 (0.65)	0.45 (6.7e-8)	−0.54 (2.1e-11)
NetShort	−0.38 (7.7e-6)	0.30 (4.8e-4)	−0.65 (6.6–17)
NetCombo	−0.22 (0.01)	0.31 (2.5e-4)	−0.56 (4.9e-12)
Func. Flow	−0.07 (0.43)	0.49 (1.8e-9)	−0.46 (2.4e-8)
PageRank	−0.10 (0.24)	0.53 (8.3e-11)	−0.58 (4.0e-13)
Random Walk	−0.14 (0.12)	0.53 (4.4e-11)	−0.51 (5.1e-10)
Network Prop.	−0.31 (2.5e-4)	0.43 (3.5e-7)	−0.55 (1.3e-11)

### Dependence on the quantity of disease-associated genes

**Figure 2:**
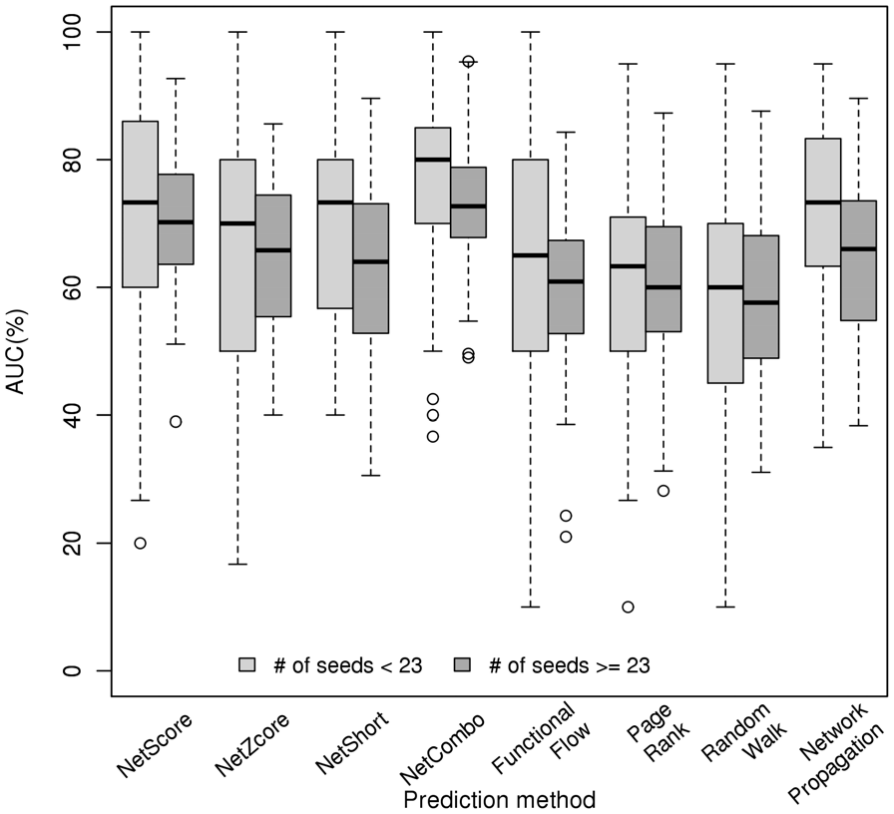
Dependence on the number of seeds. Tests and evaluations were performed using the human bPPI network and genes from OMIM, Chen and Goh disease phenotypes. Box plots of the AUCs are based on the predictions of disease-gene associations for disorders with less than 23 seeds (light gray) and disorders with at least 23 seeds (dark gray) using each prioritization method.

We questioned whether our methods depended on the number of seeds associated with a disorder using OMIM, Goh and Chen data sets. We addressed the dependence on the number of seeds by splitting all disorders into two groups with respect to the number of seeds (i.e. using the median of the distribution of the seeds associated with the diseases). There were 65 disorders with less than 23 seeds (the median number of seeds) and 67 disorders with at least 23 seeds (2 disorders had exactly 23 seeds). Figure 2 shows the AUC distribution for the eight methods studied for these two groups using bPPI network. In general, the AUCs were similar in the two groups, supporting the lack of correlation between the number of seeds and AUC in Table 2. The differences between AUCs of the two groups were only significant for NetCombo, NetShort and Network propagation (all associated p-values are less than *0.009*, assessed by non-paired Wilcoxon test). This was consistent with the anti-correlation observed between the number of seeds and AUC for these methods.

### Investigating the prioritized genes in Alzheimer's Disease, diabetes and AIDS

Using disease-gene association information in OMIM data set and the proposed consensus prioritization method (NetCombo) on the human interactome, we calculated the disease-association scores of all genes in the network for Alzheimer's Disease (AD), diabetes and AIDS, three phenotypes with relatively high prevalence in the society. In order to check the validity of these scores, we used disease-gene associations from the Comparative Toxicogenomics Database (CTD) [38], the Genetic Association Database (GAD) [39] and available expert curated data sets (see Methods for details). Moreover, we analyzed the GO functional enrichment of the top-ranking genes.

**Figure 3:**
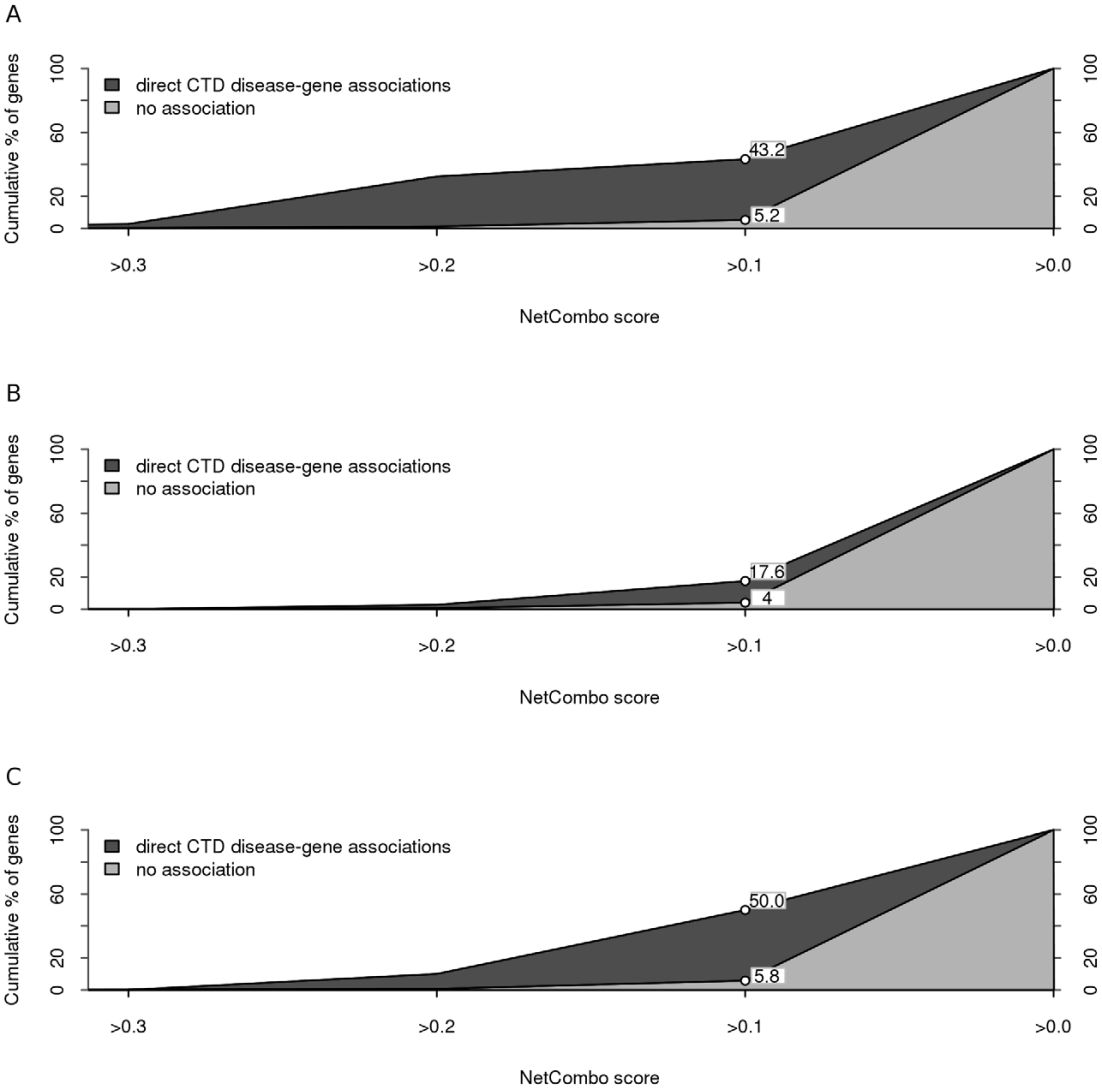
Cumulative percentage of disease-genes with direct associations in CTD (dark gray) and non associated genes (light gray) as a function of the NetCombo score for Alzheimer's disease (A), diabetes (B), and AIDS (C).

First, we used the disease-gene associations in CTD [38] to confirm the biological significance of the scores calculated by the prioritization method in these three diseases. We retrieved direct and indirect disease-gene associations in CTD. We compared the distribution of the scores assigned by NetCombo in the “direct association group” with the distribution of these scores in the “no-association group” and with the distribution in the “indirect association group” (see methods for details). In the three examples, the scores were significantly higher for the direct disease-gene associations than indirect-associations or no-associations (see Figure 3 and Table S6). In the analysis of AD and AIDS, more than 40% of the CTD disease-genes had NetCombo score higher than *0.1*. Moreover, only around 5% of the genes in the no-association group for each disease had scores higher than *0.1* and the mean of the direct association group was significantly higher than the mean of the indirect association group (Table S6).

Second, we checked how many of the gene-disease associations in GAD coincided with the top-ranking genes for each phenotype (AD, diabetes and AIDS). The top-ranking genes covered significant number of genes in GAD (Table 3). The rankings of the highest scoring genes for AD, diabetes and AIDS are given in Table S7. Then, we checked the GO functions enriched among the top-ranking genes (Table S8). GO enrichment in the subnetwork induced by the top-ranking genes in AD highlighted the role of the *Notch signaling* and *amyloid processing* pathways. The link between these pathways and the pathology of AD has been demonstrated recently [40]. The enrichment of GO functions among the prioritized genes for AIDS and diabetes showed the relevance of biological process triggered by inflammatory response, such as cytokine and in particular chemokin activity. This result was also consistent with the literature [41], [42].

**Table 3:** Number of genes (excluding seeds) in the top 1% using NetCombo score and its significance with respect to the number of genes in GAD and in the network.

	Number of top-ranking genes (predictions)	Number of top-ranking genes in GAD	Number of GAD genes in the network	Number of genes in the network	P-value (*P≤p*)
AD	89	13	107	9469	1.6e-11
Diabetes	56	5	183	9432	4.5e-03
AIDS	102	3	11	9477	1.9e-04

**Figure 4:**
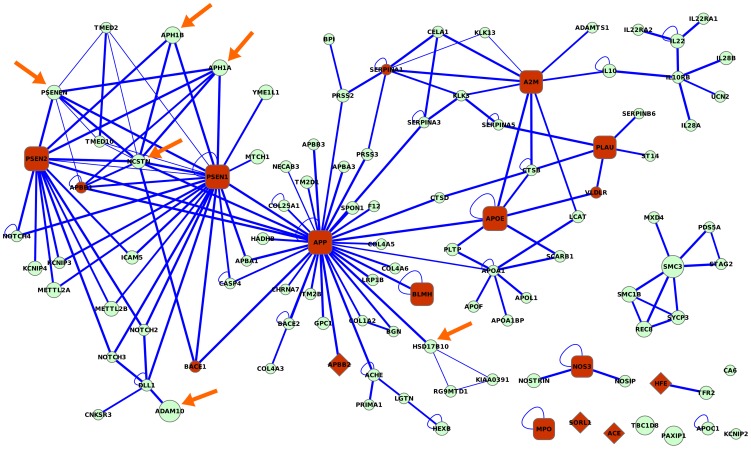
Alzheimer's disease-associated top-scored proteins and their interactions. AD-implicated proteins identified using NetCombo method on the weighted bPPI network with OMIM AD data. High-scored proteins were selected at the top 1% level using NetCombo scores. Proteins are labeled with the gene symbols of their corresponding genes. Edge thickness was proportional to the weight of the edge (assigned with respect to STRING score). Red nodes are associated with AD. Diamond and round rectangle nodes come from the OMIM AD set (seeds). Round rectangle and red circle nodes have been associated with AD using the analysis of differential expression. The nodes highlighted with arrows (*ADAM10*, *HSD17B10*, *PSENEN*, *APH1A*, *APH1B*, *NCSTN*) have been recently reported in the literature to be involved in the pathology of AD.

Finally, we further analyzed in detail the results for AD, showing that some well-ranked top genes were out of any known linkage interval associated with AD and still played a relevant role. Figure 4 shows the top-scoring genes for AD and the subnetwork induced by the interactions between their proteins. The 17 AD seeds (disease-gene associations from OMIM) and the 106 genes prioritized by NetCombo involved several protein complexes and signaling pathways such as the gamma-secretase complex, serine protease inhibitors, the cohesin complex, structural maintenance of chromosome (SMC) family, the short-chain dehydrogenases/reductases (SDR) family, adamalysin (ADAM) family, cytokine receptor family and Notch signaling pathway. Some genes within these families have been demonstrated to be involved in AD pathology [43]–[45]: ADAM10 (ADAM family), *HSD17B10* (SDR family), and *PSENEN*, *APH1A*, *APH1B*, and *NCSTN* (gamma-secretase complex). It is worth mentioning that AD has been central to recent research efforts, but mechanisms underlying the disorder are still far from understood. The accumulation of senile plaques and neurofibrillary tangles is postulated as the main cause of the disease. The gamma-secretase is involved in the cleavage of the amyloid precursor protein. This process produces the amyloid beta peptide, the primary constituent of the senile plaques in AD. Interestingly, the six genes predicted by the method (pointed by arrows in Figure 4) were not associated with AD in OMIM. Remarkably, only *APH1A (1q21–q22)*, and *PSENEN (19q13.13)* lied either under or close to a linkage interval associated with AD (i.e. *1q21*, *OMIM:611152*; and *19q13.32*, *OMIM:107741*) and none of the remaining four genes lied under or close to a known linkage interval associated with AD. Moreover, the subnetwork of top-ranking AD genes covered several genes in the expert curated data set reported by Krauthammer et al. [46] such as *APBB1*, *VLDLR*, *SERPINA1 and BACE1* (p-value associated with this event<*1.3e-3*).

## Discussion

The main contributions of this paper are twofold. First, we presented four novel methods that are comparable to, or outperform, state-of-the-art approaches on the use of protein-protein interactions to predict gene-phenotype associations at genome-wide scale, extending the set of relevant genes of a phenotype. Second, we demonstrated to which extent these prioritization methods could be used to prioritize genes on multiple gene-phenotype association and interaction data sets.

We investigated the prediction capacity and robustness of the approaches by testing their performance against the quality and number of interactions. Typically, network-based methods consider the paths between nodes equally relevant for a particular disease. The prioritization methods proposed in this study differ from others in the way the information is transferred through the network topology. NetShort considered a path between nodes shorter if it contained more seeds (known-disease gene associations) in comparison to other paths. NetScore accounted for multiple shortest paths between nodes. NetZcore assessed the biological significance of the neighborhood configuration of a node using an ensemble of networks in which nodes were swapped randomly but the topology of the original network was preserved. Our results demonstrated that combining different prioritization methods could exploit better the global topology of the network than existing methods. The prediction performance of the prioritization methods depended on the quality and size of the underlying interaction network. Yet, this dependence affected the performance of the methods similarly. The improvement of the network quality also improved the predictions for all methods. On the other hand, the prediction accuracy of the prioritization methods showed a large variation depending on the phenotype in consideration, but this variation was reduced when a consensus method was used (NetCombo).

On average, the prediction performance was better on Chen and OMIM data sets compared to the Goh data set. It can be argued that this is because the Goh data set contains gene-phenotype associations where the phenotype is defined in a broader sense (i.e. the physiological system affected). Still, the AUC values were consistent among different data sets for all the prioritization methods. Although network-based prioritization of whole genome provides a ranking of genes according to their phenotypic relevance, the interplay between genes in many diseases might not be captured by solely the PPI information. In fact, for several phenotypes in OMIM data set such as *amyloidosis, myasthenic, myocardial* and *xeroderma* the genes associated with the disease were predicted with high accuracy in our analysis, whereas for *mitochondrial*, *osteopetrosis* and *epilepsy* phenotypes, the network-based prioritization was less successful.

The best AUC and coverage of disease genes among high-scored gene-products were obtained with the largest and highly confident network (in which interactions integrated from public repositories were filtered out if detected by TAP and edges were positively weighted using the scores provided by STRING database). This improvement was significant for all proposed approaches. The increased coverage and AUC when the bPPI network was used instead of the Goh and Entrez networks showed the benefit of integrating information from various data sources. Prioritization algorithms rely on the topology of the network; thus, increasing the number of known interactions should improve coverage. Nonetheless, interaction data integrated in this manner is prone to include false positives, and filtering possible non-binary interactions (e.g., complexes identified by TAP) can improve the use of integrated data. The hypothesis that we required the largest reliable set for the study of gene prioritization was supported by the increase of AUC when the bPPI network was used instead of the PPI network.

The AUC values over dozens of different phenotypes that vary in number of initial gene-phenotype associations showed the applicability of the methods independent of the number of genes originally associated with the phenotype. Moreover, having more number of seeds associated with a pathophenotype did not necessarily improve the prediction accuracy. Most prioritization methods achieved better performance for disorders with low number of seeds. This difference in performance was significant for NetCombo, NetShort and Network propagation. In fact, the accuracy of the predictions was rather correlated with the average shortest path length between seeds, which shows the importance of the topology of the network.

We applied the prioritization methods to study the implication of genes in AD, diabetes and AIDS. We claimed that the genes discovered in the high scoring portion of the network would be more likely to be involved in the pathology of these diseases. Therefore, we further analyzed the genes prioritized by NetCombo using the human bPPI network. We verified that some of these predictions were consistent with the literature and the scores assigned by GUILD distinguished between the genes associated with a specific disease and the rest of genes. We have to note that we merged the entries for diabetes type 1 and type 2 in OMIM and defined it as “diabetes phenotype”. This may explain why 1) the top-ranking genes predicted for diabetes covered relatively less genes in GAD (assessed by hypergeometric p-value) than AD and AIDS; and 2) the genes with direct-associations were more easily segregated by NetCombo-scores for AD and AIDS than diabetes. Furthermore, we showed that the groups of genes predicted to be associated with these three phenotypes were enriched in biological processes related to the disease. In AD, top-ranking genes formed a subnetwork implying the Notch and amyloid pathways, while top-ranking genes for diabetes and AIDS were involved in the inflammatory response mechanisms. Our analysis on these diseases suggested that our approach in whole genome prioritization was a competent way to discover novel genes contributing to the pathology of diseases.

Based on this study, we have shown that the new approaches (NetCombo, NetShort, NetScore, and NetZcore) improved the results of state-of-the-art algorithms, such as Functional Flow, PageRank with priors, Random walk with restart and Network propagation. It is worth mentioning that PageRank with priors and Random walk with restart have been adopted to address genome-wide disease-gene prioritization previously [24], [28]. Furthermore, a variation of Random walk with restart algorithm that incorporates phenotypic similarity was recently proposed [47]. Since our aim was to compare the algorithms with each other, here, we evaluated them on the same benchmarking data set using only the initial disease-gene associations and the interaction network. Finally, we made all eight methods publicly available in the GUILD framework.

Overall, our results suggest that human diseases employ different mechanisms of communication through their interactions. Our analysis reveals a collective involvement of sets of genes in disorders and could be extended to identify higher order macromolecular complexes and pathways associated with the phenotype. However, the use of a single and generic prioritization scheme may not be sufficient for completing the set of pathways affected by a disease and may require the use of more than one method. Furthermore, network-based prioritization methods that use only PPI information fail to identify the disease-genes whose proteins do not interact with other proteins. Therefore, towards a comprehensive understanding of biological pathways underlying diseases, the network-based prioritization methods suggested here can be complemented by incorporating gene expression, functional annotations or phenotypic similarity profiles and by using functional association networks rather than PPI networks.

## Methods

### Protein-protein interaction data sets

We used three human interactomes: *i) Goh network*, the PPI network from the work of Goh et al. [13] in which data was taken from two high quality yeast two-hybrid experiments [48], [49] and PPIs obtained from the literature; *ii) Entrez network*, a compilation of interactions from BIND and HPRD provided by NCBI (ftp://ftp.ncbi.nih.gov/gene/GeneRIF/interactions.gz); and *iii) PPI network*, the set of experimentally known PPIs integrated as in Garcia-Garcia and colleagues using BIANA [50] (see Methods S1 on the details of the integration protocol). Considering that high throughput pull down interaction detection methods introduce many indirect relationships (such as being involved in the same complex) in addition to direct physical interactions, we removed the subset of interactions obtained by TAP, resulting in the *bPPI network*. Furthermore, we have incorporated edge scores for the interactions between two proteins in this network using STRING database [37]. We refer this network as *weighted bPPI network*. In all other networks, the edge weights have the default value of *1*. When edge weights from STRING were used (in weighted bPPI network), the scores given by STRING were rescaled to range between *0* and *1* and then added to the default value of *1*.

We have to note that the algorithms being studied depend solely on the topology of the network, implying that unconnected nodes and very small components cannot effectively transfer the relevant information along the network. Consequently, only the largest connected component of the network was used for the evaluation (see Table S9 for the sizes of the remaining components in the interaction networks). Hereafter, the term “network” refers to the largest connected component of the network unless otherwise stated. See Table S10 for a summary of the data contained in these interaction networks.

### Gene–phenotype associations

Genes and their associated disorders were taken from: 1) Online Mendelian Inheritance in Man (OMIM) database [51], 2) Goh et al. [13] (referred as Goh data set throughout the text), and 3) Chen et al. [52] (referred as Chen data set throughout the text). OMIM is one of the most comprehensive, authoritative and up-to-date repositories on human genes and genetic disorders. The information in OMIM is expert curated and provides the mutations on the genes associated with the disorders. Phenotypic associations for genes were extracted from the OMIM Morbid Map (omim.org/downloads retrieved on November 4, 2011) by merging entries using the first name as previously done [15], [19], [24]. A disorder was considered if and only if it had at least 5 gene products in any of the interaction networks mentioned above (this data set is referred as OMIM hereafter). Having 5 proteins in the interaction network was required for a five-fold cross validation evaluation and also ensured that we tested the capacity to use global topology (in the case of few genes the amount of annotation transfer is limited, diminishing the benefit of using network based methods as opposed to direct neighborhood). In Goh data set [13], OMIM disorders (from December 2005) were manually classified in 22 disorder classes based on the physiological system affected (21 classes excluding the unclassified category). In Chen data set [52], a total of 19 diseases were collected from OMIM and GAD See Table S11 for a summary of the diseases used in this study.

Additionally, we used an independent gene-phenotype association data set to optimize the required parameters of prioritization methods (see below) without over-fitting the available gene-disease associations. This data set contains gene-disease associations identified by text mining PubMed abstracts using SCAIView [53] for aneurysm (168 genes, keyword search “*intracranial aneurysm*” and restricting the query to include entries with MeSH “*genetics*” term) and breast cancer (1588 genes, similar to aneurysm but using “breast cancer” as the keyword). These genes are listed in Table S12.

Genes associated with a disorder were mapped to their products (proteins) in the protein-protein interaction network and assigned an initial score for their phenotypic relevance. Thus, proteins translated by genes known to be involved in a particular pathology were termed *seeds* and have the higher scores in the network. All other proteins in the network were assigned *non-seed* scores (lower scores in the network).

The number of proteins (nodes) and interactions (edges) in all interaction networks used in this study are given in Table S10. Table S11 summarizes all diseases used under the context of this study, the number of genes associated with them and number of corresponding proteins translated by these genes covered in the largest connected component of the network.

### Network based prioritization algorithms

***NetShort*** is motivated by the idea that a node important for a given phenotype would have shorter distances to other seed nodes in the network. As opposed to previous approaches that employ shortest paths, we incorporate “disease-relevance” of the path between a node and disease nodes by considering not only the number of links that reach to the disease-associated node but also number of disease-associated nodes that are included in the path. Thus, we modify the length (weight) of the links in shortest path algorithm such that the links connecting seed nodes are shorter than the links connecting non-seed nodes. Formally the score of a node, u, is defined as:
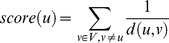
where *d(u,v)* is the shortest path length between nodes *u* and *v* with weighted edges of graph *G(V,E,f)*. The graph is defined by nodes *V*, edges *E*, and the edge weight mapping function, f, where f is defined as 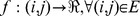. The weight *f(i,j)* is given by the multiplication of edge score and average of the initial scores of both nodes as follows:
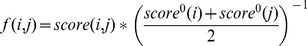
This definition implies that the edge is short when the scores of the nodes forming the edge are high (e.g. when they are seeds) and long otherwise.

***NetZcore*** assesses the relevance of a node for a given phenotype by normalizing scores of nodes in a network with respect to a set of random networks with similar topology. Intuitively, NetZcore extends the direct neighborhood approach, where all the neighbors of the node contribute to the relevance of the node, to a normalized direct neighborhood. It highlights the relevance of the node compared to the background distribution of the relevance of neighboring nodes (using random networks). The score of a node is calculated as the average of the scores of its neighboring nodes. This score is then normalized using the z-score formula:
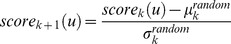
where 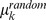 and 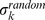 are the mean and standard deviation of the distribution of scores in a set of random networks with the same topology as the original graph. Networks with the same topology are generated such that a node *u* having degree *d* is swapped with another node *v* in the network with the same degree *d*. In this study, we use a set of *100* random networks. The process of calculating node scores based on the neighbor scores using random networks is repeated by a number of times (iterations) specified by the user in order to propagate the information along the links of the network. The iteration number (*k*) varies from *1* to a maximum (*MaxZ*). *MaxZ* is a specific parameter of the method, and *score_k_(u)* at iteration k is calculated as:
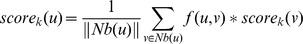
where in a graph *G(V,E,f)* with nodes *V*, edges *E*, *Nb(u)* is the set of neighbors of node *u*, and *f(u,v) = weight(u,v)* is an edge weight mapping function. Note that, NetZcore incorporates the statistical adjustment method suggested by Erten and colleagues into the scoring by both normalizing and propagating scores at each iteration [25].

***NetScore*** is based on the propagation of information through the nodes in the network by considering multiple shortest paths from the source of information to the target and ignoring all other paths between them. To calculate the information passed through all the shortest paths in between two nodes, NetScore uses a message-passing scheme such that each node sends its associated information as a message to the neighbors and then iteratively to their neighbors (pseudo-code is given in Figure S3). Each message contains the node identity of the emitter and the path weight (defined as the multiplication of edge weights of the path that the message has traveled). Messages are stored in each node so that only the first messages arriving from a node are considered (i.e. the messages arriving through all the shortest paths from that node). At the end of each iteration, the score of a node is defined as the average score for the messages received. The score carried by a message is calculated as the score of the emitter multiplied by the path weight. Thus, at iteration k, a node has the score of the nodes reaching it from shortest paths of length k (more than once if multiple shortest paths exist) weighted by the edge weights in these paths. Considering that storing all the messages coming from the k-neighborhood introduces a memory and time penalty, we restrict the number of iterations during score calculation to a maximum (*MaxS*). To cover the whole diameter of the network, we repeat the scoring with updated scores after emptying the message arrays (resetting the node scores with the scores accumulated in the last iteration). Therefore, in addition to the number of iterations (*MaxS*), NetScore uses the number of repetitions (*NR*) as parameters of the algorithm.

***NetCombo*** combines NetScore, NetShort and NetZcore in a consensus scheme by averaging the normalized score of each prioritization method. The normalized score of a prioritization method for a node *n* is calculated using the distribution of scores with this method. The mean of the scores of all nodes prioritized by this method is subtracted from the score of node *n* and then divided by the standard deviation of the distribution.

In addition to the four methods above, four state-of-the-art algorithms have been included in GUILD for prediction performance comparison purposes. These methods are PageRank with priors [35] (as used in ToppNet [23]), Functional Flow [36], Random walk with restart [19] and Network propagation [22]. See Methods S1 for the details of the implementation of these methods. PageRank with priors has recently been proven to be superior to available topology-based prioritization methods [24], [28]. The methods based on random walk with restart proposed by Kohler et al. [19] and propagation algorithm by Vanunu et al. [22] are both conceptually similar to PageRank with priors and differ in the way that they incorporate link weights (edge scores) [22], [25]. We also apply Functional Flow, a global network topology-based method, originally addressed the functional annotation problem [36].

### Prediction performance evaluation

To evaluate the prioritization methods, we used five-fold cross validation on three gene-phenotype annotation data sets mentioned above. Proteins known to be associated with a phenotype (seeds) were split into five groups; four of them were used as seeds for the prioritization methods and the remaining one group was used to evaluate the predictions. This process was repeated five times, changing the group for evaluation each time. The area under the ROC curve (AUC) and sensitivity were averaged over the five folds. These averages and their standard deviations were used to assess the quality of the predictions and compare the methods. A ROC (receiver operating characteristic) curve plots true positive rate (sensitivity) against false positive rate (*1*-specificity) while the threshold for considering a prediction as a positive prediction is varied. The AUC is the area under this plot and corresponds to the probability that a classifier will rank a randomly chosen positive instance higher than a randomly chosen negative one. ROCR package [54] was used to calculate these performance metrics and the selection of positive and negative instance scores are explained in the next paragraph.

In the context of functional annotation and gene-phenotype association studies, obtaining negative data (proteins/genes that have no effect on a disease, disorder, or phenotype) is a challenge. We tackled this problem with an alternative procedure. First, all proteins not associated with a particular disease (or phenotype) were treated as potential negatives. Then, we used a random sampling (without replacement) of the potential negatives to calculate an average score. This score was defined as the score of a *negative instance*. We calculated as many scores of *negative instances* as *positive instances* (seeds) in the evaluation set. We ensured that each of the potential negatives were included in one of the random samples by setting the sample size equal to the number of all potential negatives divided by the number of seeds. Using this procedure, we had the same number of positive and negative scores, and the probability associated with choosing a positive instance by chance was *0.5*.

We used the aforementioned data sets for aneurysm and breast cancer to optimize the initial scores of seeds and non-seeds and the following parameters of the prioritization methods: MaxZ for NetZcore, MaxF for Functional Flow, and MaxS and NR for NetScore. For each of these parameters, the values that result in the largest average five-fold cross validation AUC were selected. The optimal values for initial scores of seeds and non-seeds were identified as *1.00* and *0.01* respectively, among the values we have tested (*1.00* or text mining score associated with the seed, for seeds; and *0.01*, *1.0e-3*, *1.0e-5* or *0*, for non-seeds). The number of iterations for NetZcore (MaxZ) and Functional Flow (MaxF) was *5*. In the case of Functional Flow, *5* was also the limit specified by the authors. For NetScore, the optimized values were two iterations (MaxS) with three repetitions (NR).

To test the significance of AUC differences between a pair of networks, or prioritization approaches, the one-sided Wilcoxon test was used. The alternative hypothesis was that the mean AUC of the network (or prioritization method) under consideration was greater than the other network under test (or prioritization method). No assumption was made regarding the normality of the distribution of AUCs, and AUCs were paired over the variable in concern (either network type or prioritization method); thus, a non-parametric paired test was applied. Alpha values were set to *0.05*. The values for the samples of the random variable subject to the statistical test are given in relevant supplementary tables. R software (http://www.r-project.org) was used to compute statistics.

### Assessing seed connectivity in the network

We investigated the relationship between prediction performance of the prioritization methods and the connectivity of seeds in the network. We calculated the average number of neighbor seeds and the average shortest path distances between each pair of seeds for each phenotype as in Navlakha and Kingsford [24]. The average number of neighbor seeds (*Ns*) is given as follows:
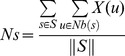
where *S* is the set of seeds, *Nb(s)* is the set of nodes interacting with s (neighbors), and *X(u)* is *1* if *u* belongs to *S* and *0*; otherwise. Similarly, the average shortest path distances (*Ss*) are given by
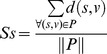
where 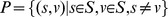 is the set of all seed pairs and *d(s,v)* is the shortest distance between *s* and *v*.

### Analysis of prioritized genes in Alzheimer's disease, diabetes and AIDS

We used the weighted bPPI network and products of AD, diabetes and AIDS seeds according to OMIM to investigate high-scoring nodes (top 1%) obtained with NetCombo algorithm. We calculated the scores by applying NetCombo and then selected 113 proteins in the network (top 1% of 11250 proteins in the network). These proteins were uniquely mapped to their corresponding gene symbols, yielding 106, 110 and 109 genes for AD, diabetes and AIDS respectively. Next, we counted how many of these genes were listed in Genetic Association Database (GAD) [39] for each phenotype. GAD is a database that catalogs disease-gene associations curated from genetic association studies and collects findings of low significance in addition to those with high significance. We considered only the records in GAD that reported a positive association and merged the entries using the first name of the disease as we did for OMIM data set. In this analysis we excluded the seeds (disease-gene associations in OMIM). The p-values shown in Table 3 correspond to the probability of identifying GAD disease-gene associations at the top-ranking portion of the network assuming a hypergeometric model. The level of significance was set to *0.05*. For AD, we also checked whether the top-ranking genes covered the expert curated genes implicated in AD pathology reported in Krauthammer et al. [46].

We analyzed the GO functional enrichment of the top-ranking genes using FuncAssociate2.0 [55] web service. The background consisted of all the genes in the network. A GO term was associated with a gene set if the adjusted p-value associated with the term was lower than *0.05*.

We used the disease-gene associations in Comparative Toxicogenomics Database (CTD) [38] to check the biological significance of the scores calculated by the prioritization method of AD, diabetes and AIDS. CTD contains both manually curated disease-gene associations (direct) and inferred disease-gene associations (indirect). Again, the entries were merged using the first name of the disease. The scores of the direct disease-genes, indirect disease-genes and no-association genes (not found in CTD) were grouped as direct-association group, indirect-association group and no-association group. We tested the difference between the means of the distributions of scores using one tailed Student's t-test (assuming higher score for the direct associations and the alpha value was set to *0.05* as before).

## Supporting Information

Methods S1 **Supplementary methods.**
(PDF)

Figure S1 **Comparison of the significance in prediction performance between prioritization methods.** Significance of the differences in average AUC performance (averaged over all interaction networks and disease data sets) is represented as a heatmap. Dark blue color in a cell (i, j) of the heatmap denotes that the p-value associated with the one sided Wilcoxon test for the comparison of AUCs between i^th^ and j^th^ method (where the alternative hypothesis is that the mean of the first is greater than the second) is smaller or equal than 0.05.
(TIF)

Figure S2 **Ratio of successful predictions among the top 1% scores obtained by each method on each interaction network over all phenotypes of OMIM, Goh and Chen data sets.** Color legend is same as Figure 1 in the manuscript.
(TIF)

Figure S3 **Pseudo-code of the NetScore algorithm.** The repetition part is handled inside the first for-loop where message arrays are reset. The inside for-loop goes over the iterations, where only “new” messages are accepted. At the end of each iteration, the score of a node is calculated based on the messages it received.
(TIF)

Table S1 **Average AUC of the prioritization methods on each data set of seeds (OMIM, Goh and Chen) using different interaction networks (Goh, Entrez, PPI, bPPI and weighted bPPI).**
(DOC)

Table S2 **P-values associated with the paired Wilcoxon signed rank test between Network Propagation and our two best prioritization methods on each data set using average AUCs over all networks.**
(DOC)

Table S3 **AUC of the prioritization methods for each disorder and network.**
(XLS)

Table S4 **Sensitivity values at top 1% predictions of the prioritization methods for each disorder and network.**
(XLS)

Table S5 **Five-fold AUC (%) for each method averaged over all diseases within the data set and all interaction networks considering all non-seeds (genes not associated with the diseases) as negatives.**
(DOC)

Table S6 **The average NetCombo scores (the standard deviation is given in parenthesis) of CTD direct/indirect disease-genes and the genes with no-association in CTD and the p-value associated with the difference between these groups.**
(DOC)

Table S7 **Top ranking genes in Alzheimer's Disease (AD), diabetes and AIDS identified by NetCombo (the top 1% high scoring genes) using weighted bPPI network and OMIM associations.**
(XLS)

Table S8 **Functional enrichment of high scoring common genes in NetCombo for AD, diabetes and AIDS.**
(XLS)

Table S9 **Number and size of the connected components other than the largest connected component (LCC) in the network.**
(DOC)

Table S10 **Interaction data sets used in the analysis.**
(DOC)

Table S11 **Number of disease-gene associations covered in each network.**
(DOC)

Table S12 **Genes used for parameter optimization.**
(DOC)
