# Supplementary material for: Exploiting Protein-Protein Interaction Networks for Genome-Wide Disease-Gene Prioritization
Source: PLoS One. 2012 Sep 21;7(9):e43557. doi: 10.1371/journal.pone.0043557 (PMC3448640; doi:10.1371/journal.pone.0043557)
Supplement: Methods S1 — Supplementary methods. (PDF) [file pone.0043557.s001.pdf]

Supplementary methods for "Guney E. and Oliva B., Exploiting Protein-Protein Interaction Networks for Genome-wide Disease-Gene Prioritization".

Integrating protein protein interactions using BIANA

BIANA (Biologic Interactions And Network Analysis), a software developed by Garcia-Garcia and colleagues [1], integrates various biological data repositories providing information on biological entities (genes, RNAs, proteins, domains, etc...) and their relationships (such as protein-protein interactions and complex involvement). BIANA unifies biological data spread over multiple data sources based on so called "*unification protocol*". Unification protocol consists of a set of rules that determine how entries in various data sources are combined into groups of "*equivalent entries*". Two entries from two different databases will be considered equivalent provided that they share the same annotation for the specified attributes in the rules. These groups of equivalent entries inherit all the attributes of all entries included in the group.

Using BIANA, we unified data in UniProt [2], KEGG [3], HGNC [4], IPI [5], Reactome [6], STRING [7], DIP [8], HPRD [9], IntAct [10], MIPS [11] and BioGRID [12] with respect to the following criteria of equivalence: all entries coming from these biological data repositories are grouped together if and only if they share *UniprotAccession* code or, both *sequence* and *Taxonomy* identifier, or *GeneID*. If an entry in concern does not share any of these annotations, a new group is created for that entry provided that it does not belong to an interaction database (Reactome, STRING, DIP, HPRD, IntAct, MIPS, BioGRID) since these databases are mainly dedicated to describe interactions rather than biological entites and may provide confounding identifiers. Once equivalent entries are created using this unification protocol, we created an interaction network by first querying BIANA database for all groups of entries associated with "Homo sapiens" using 9606 as *Taxonomy* identifier (nodes of the network) and then by getting all the interactions given for these groups of entries (edges of the network). Having such groups of equivalent entries provides a way to map protein-protein interaction data provided in different databases, typically with non-uniform identifiers (using different nomenclature). On the other hand, due to ambiguous or incomplete annotations in databases, this unification approach is prone to creating multiple equivalent entry groups with similar annotations. If the values of the attributes used in the unification protocol differ for a pair of entries, they will not be grouped together although they share annotations for other attributes. For example, say a database A refers the product of gene "EMR1" by *UniprotAccession* of "Q14246", no GeneID and a sequence S whereas another database B refers it with *GeneID* of "2015", no *UniprotAccession* and a sequence S' (differing from S by only a few amino acids). Then according to the unification protocol above, there will be two groups of entries corresponding to two very similar protein products of the same gene "EMR1".

Existing prioritization methods included in the evaluation

Functional Flow [13] is based on the idea of spreading scores over a network from annotated nodes towards non-annotated nodes. At each iteration, annotation scores flow from nodes with a higher score to nodes with lower scores in an amount equivalent to the capacity of the edge through which the nodes are connected. We based our implementation on the original method described in Nabieva et al. [13] . The total number of iterations (*MaxF*) is a parameter for this method that needs to be optimized.

PageRank with priors algorithm [14,15] adopts a random walk based model to score a node in terms of phenotypic relevance, in which a random surfer is more likely to end up in initially relevant nodes. Application of the method in candidate disease gene prioritization was proposed by Chen et al. [15]. Similar to their implementation, in the GUILD framework, interactions in the network are treated as

bidirectional links and node association scores are assigned using the formula:

$$PR_{t+1}(u) = (1-d) * PR_0(u) + d * \sum_{(u,v)\in E} \frac{weight(u,v) * PR_t(v)}{\|\{(u,v)\in E\}\|}$$

where u is the current node in consideration, v is a node linked with u, d is a *damping factor* (probability that the process of following the links will continue), V and E the nodes and edges, respectively, and $PR_t(u)$ is defined as the page-rank of node u at step t, which is updated iteratively. A node has an initial page-rank value of *1* if it is a seed and *0.01* otherwise. These initial page-rank values are then normalized such that $\sum_{u\in V} PR_0(u) = 1$.

The damping factor is set to *0.15*. However, this value has little effect on the prediction as long as it is in the interval *[0.1,0.3]* as suggested by the authors. The implementation of PageRank used by the authors is publicly available as a JAVA library. We verified that our implementation based on Boost Graph Library (http://www.boost.org) is consistent with the original implementation.

Random walk with restart [16] iteratively simulates random transitions of a walker from a node to a randomly selected neighbor node and where at any time step the walk can be restarted depending on a predefined probability. Random walk with restart is slightly different than PageRank with priors in the way that it normalizes the link weights. The scores of nodes are formally given by:

$$p_{t+1} = (1-r) * Wp_t + r * p_0$$

where p_t is a vector where each element i holds the probability of being at node i of the network at time step t, W is the column normalized adjacency matrix and r is the restart probability. p_0 contains the initial probabilities for the nodes and the sum of these probabilities is *1* (similar to initial page-rank values above). p_{t+1} contains the disease-association probabilities of nodes once the convergence is reached. The convergence is decided by either having a difference less than *10e-6* between p_{t+1} and p_t or achieving the limit of the number of iterations, set as *50* (though in practice less than 20 iterations are typically sufficient to satisfy the first criterion).

Network propagation [17] modifies Random walk with restart such that the link weight is normalized not only by number of outgoing edges but also by number of incoming edges. Following the definitions of Vanunu et al. [17] and the clarifications of Erten et al. [18], the network propagation is formulated as follows:

$$p_{t+1} = (1-r) * W' p_t + r * p_0$$

where p_t, p_0, r are all defined as before for Random walk with restart. W', on the other hand, is the adjacency matrix whose elements are normalized by the square root of the multiplication of node degrees of the nodes that define the edge at that cell.

Supplementary References

1. Garcia-Garcia JG (2010) Biana: a software framework for compiling biological interactions and analyzing networks. BMC Bioinformatics 11: 56.

2. Magrane M, others (2011) UniProt Knowledgebase: a hub of integrated protein data. Database: the journal of biological databases and curation 2011.

3. Kanehisa M, Goto S, Sato Y, Furumichi M, Tanabe M (2012) KEGG for integration and interpretation of large-scale molecular data sets. Nucleic acids research 40: D109–D114.

4. Seal RL, Gordon SM, Lush MJ, Wright MW, Bruford EA (2010) genenames.org: the HGNC resources in 2011. Nucleic Acids Research 39: D514–D519. doi:10.1093/nar/gkq892.

5. Kersey PJ, Duarte J, Williams A, Karavidopoulou Y, Birney E, et al. (2004) The International Protein Index: an integrated database for proteomics experiments. Proteomics 4: 1985–1988.

6. Croft D, O'Kelly G, Wu G, Haw R, Gillespie M, et al. (2011) Reactome: a database of reactions, pathways and biological processes. Nucleic acids research 39: D691.

7. von Mering CJ (2007) STRING 7--recent developments in the integration and prediction of protein interactions. Nucleic Acids Res 35: D358–D362.

8. Salwinski L, Miller CS, Smith AJ, Pettit FK, Bowie JU, et al. (2004) The Database of Interacting Proteins: 2004 update. Nucleic Acids Res 32: D449–51.

9. Keshava Prasad TS, Goel R, Kandasamy K, Keerthikumar S, Kumar S, et al. (2009) Human Protein Reference Database--2009 update. Nucleic Acids Research 37: D767–D772. doi:10.1093/nar/gkn892.

10. Kerrien S, Aranda B, Breuza L, Bridge A, Broackes-Carter F, et al. (2011) The IntAct molecular interaction database in 2012. Nucleic Acids Research 40: D841–D846. doi:10.1093/nar/gkr1088.

11. Mewes HW, Ruepp A, Theis F, Rattei T, Walter M, et al. (2010) MIPS: curated databases and comprehensive secondary data resources in 2010. Nucleic Acids Research 39: D220–D224. doi:10.1093/nar/gkq1157.

12. Stark C, Breitkreutz B-J, Chatr-aryamontri A, Boucher L, Oughtred R, et al. (2010) The BioGRID Interaction Database: 2011 update. Nucleic Acids Research 39: D698–D704. doi:10.1093/nar/gkq1116.

13. Nabieva EJ (2005) Whole-proteome prediction of protein function via graph-theoretic analysis of interaction maps. Bioinformatics 21: i302–i310.

14. White S, Smyth P (2003) Algorithms for estimating relative importance in networks. Proceedings of the ninth ACM SIGKDD international conference on Knowledge discovery and data mining. Washington, D.C.: ACM. pp. 266–275.

15. Chen JA (2009) Disease candidate gene identification and prioritization using protein interaction

networks. BMC Bioinformatics 10: 73.

16. Kohler S, Bauer S, Horn D, Robinson PN (2008) Walking the interactome for prioritization of candidate disease genes. Am J Hum Genet 82: 949–958.

17. Vanunu O, Magger O, Ruppin E, Shlomi T, Sharan R (2010) Associating genes and protein complexes with disease via network propagation. PLoS computational biology 6: e1000641.

18. Erten S, Bebek G, Ewing RM, Koyutürk M, others (2011) DADA: Degree-Aware Algorithms for Network-Based Disease Gene Prioritization. BioData mining 4: 19.

19. Goh KIC (2007) The human disease network. Proceedings of the National Academy of Sciences 104: 8685.

20. Rual J-FV (2005) Towards a proteome-scale map of the human protein-protein interaction network. Nature 437: 1173–1178.

21. Stelzl UW (2005) A human protein-protein interaction network: a resource for annotating the proteome. Cell 122: 957–968.

22. Bader GD, Betel D, Hogue CW (2003) BIND: the Biomolecular Interaction Network Database. Nucleic Acids Res 31: 248–250.

23. Guldener U, Munsterkotter M, Oesterheld M, Pagel P, Ruepp A, et al. (2006) MPact: the MIPS protein interaction resource on yeast. Nucleic Acids Res 34: D436–41.
