# Supplementary figures and images for: Exploiting Protein-Protein Interaction Networks for Genome-Wide Disease-Gene Prioritization
Source: PLoS One. 2012 Sep 21;7(9):e43557. doi: 10.1371/journal.pone.0043557 (PMC3448640; doi:10.1371/journal.pone.0043557)

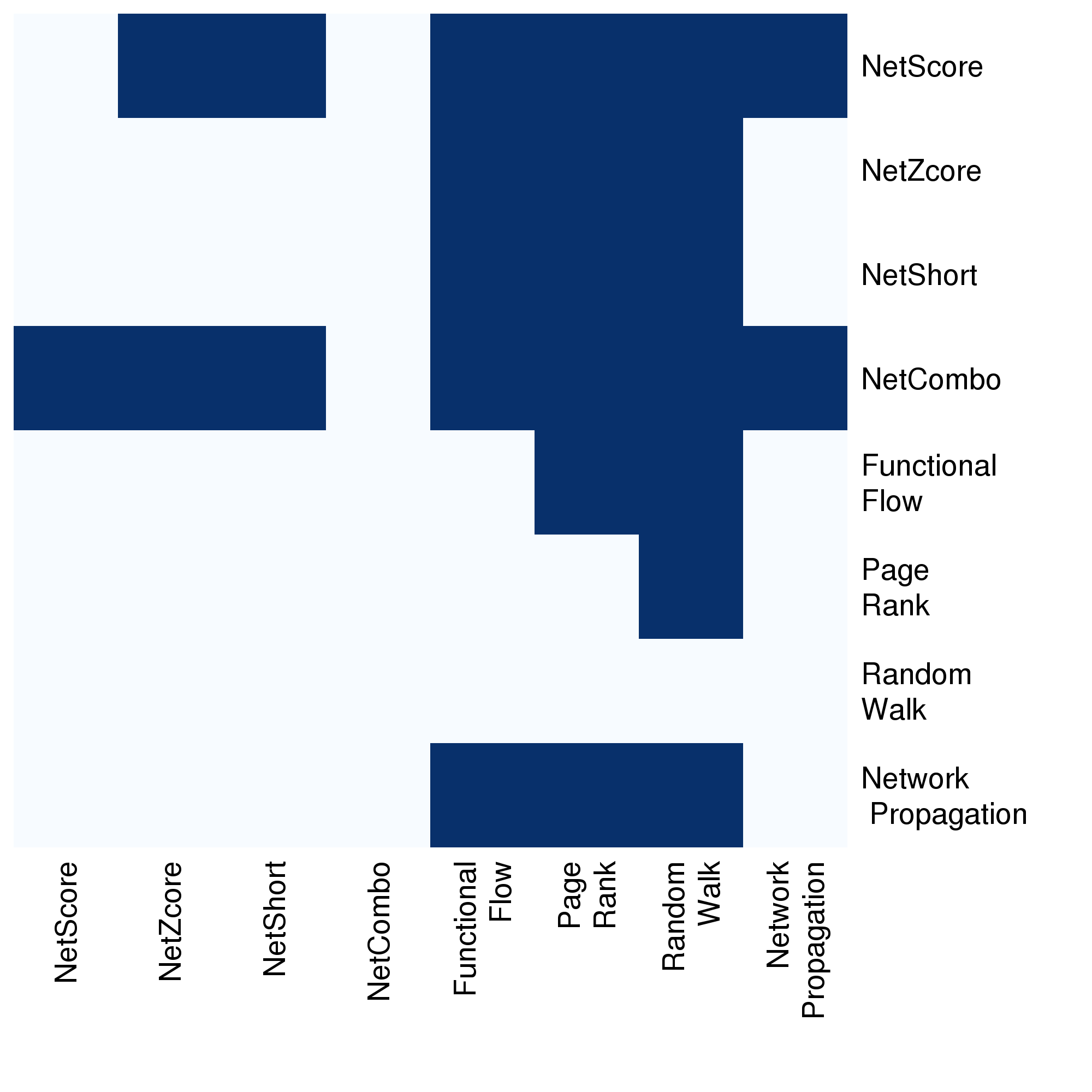

Supplement: Figure S1 — Comparison of the significance in prediction performance between prioritization methods. Significance of the differences in average AUC performance (averaged over all interaction networks and disease data sets) is represented as a heatmap. Dark blue color in a cell (i, j) of the heatmap denotes that the p-value associated with the one sided Wilcoxon test for the comparison of AUCs between ith and jth method (where the alternative hypothesis is that the mean of the first is greater than the second) is smaller or equal than 0.05. (TIF) [file pone.0043557.s002.tif]

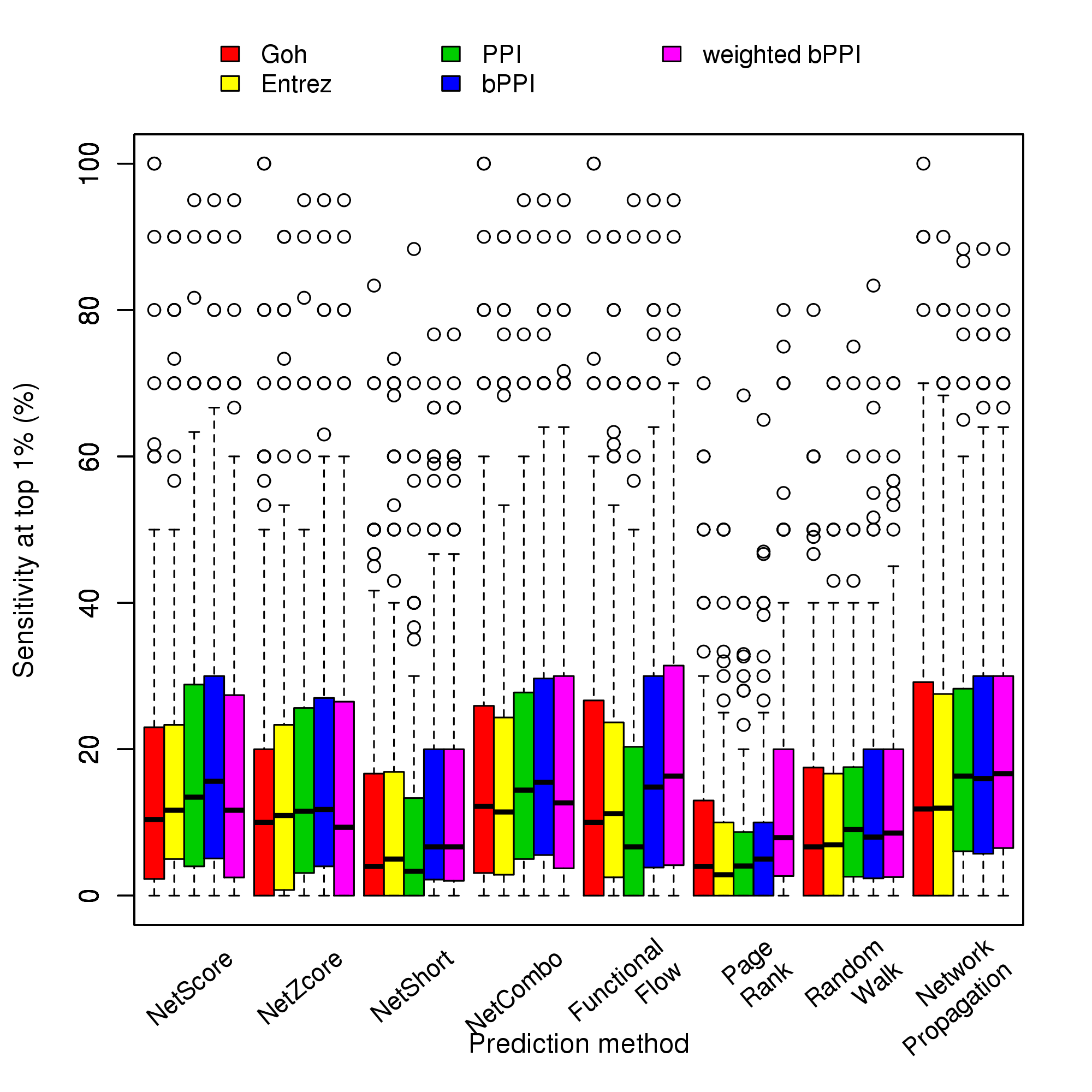

Supplement: Figure S2 — Ratio of successful predictions among the top 1% scores obtained by each method on each interaction network over all phenotypes of OMIM, Goh and Chen data sets. Color legend is same as Figure 1 in the manuscript. (TIF) [file pone.0043557.s003.tif]

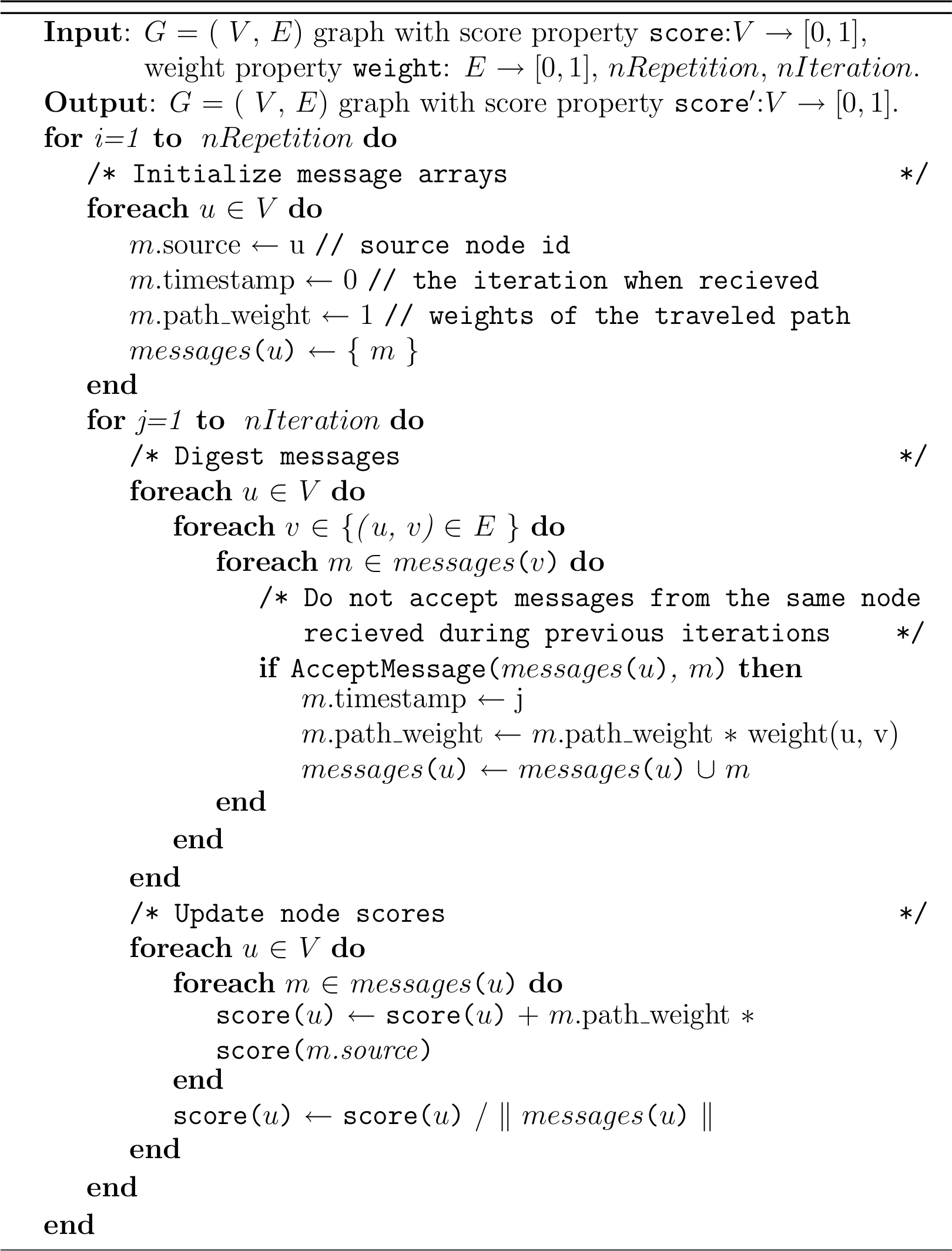

Supplement: Figure S3 — Pseudo-code of the NetScore algorithm. The repetition part is handled inside the first for-loop where message arrays are reset. The inside for-loop goes over the iterations, where only “new” messages are accepted. At the end of each iteration, the score of a node is calculated based on the messages it received. (TIF) [file pone.0043557.s004.tif]
