# Supplementary material for: Exploiting Protein-Protein Interaction Networks for Genome-Wide Disease-Gene Prioritization
Source: PLoS One. 2012 Sep 21;7(9):e43557. doi: 10.1371/journal.pone.0043557 (PMC3448640; doi:10.1371/journal.pone.0043557)
Supplement: Table S1 — Average AUC of the prioritization methods on each data set of seeds (OMIM, Goh and Chen) using different interaction networks (Goh, Entrez, PPI, bPPI and weighted bPPI). (DOC) [file pone.0043557.s005.doc]

**Table S1.**Average AUC of the prioritization methods on each data set of seeds (OMIM, Goh and Chen) using different interaction networks (Goh, Entrez, PPI, bPPI and weighted bPPI)*

| Network | Data set | NetScore | NetZcore | NetShort | NetCombo | Func. Flow | Page  Rank | Random Walk | Network Prop. |
| --- | --- | --- | --- | --- | --- | --- | --- | --- | --- |
| Goh | OMIM | 61.75 | 53.70 | 65.63 | **69.67** | 52.47 | 52.51 | 49.61 | 58.23 |
|  | Goh | 61.92 | 56.99 | 58.52 | **64.74** | 51.88 | 51.35 | 46.49 | 49.81 |
|  | Chen | 68.56 | 64.79 | 62.28 | **71.53** | 57.18 | 57.26 | 52.41 | 57.84 |
|  | Overall | 63.27 | 57.37 | 62.74 | **68.47** | 53.32 | 53.22 | 49.26 | 55.46 |
| Entrez | OMIM | 65.36 | 61.16 | 66.31 | **70.84** | 58.79 | 56.44 | 53.71 | 63.35 |
|  | Goh | **66.53** | 61.22 | 54.66 | 64.83 | 57.21 | 52.65 | 48.21 | 52.28 |
|  | Chen | 74.38 | 71.50 | 63.35 | **76.91** | 62.26 | 63.09 | 57.62 | 65.95 |
|  | Overall | 67.66 | 63.76 | 62.43 | **70.58** | 59.07 | 56.91 | 52.99 | 60.83 |
| PPI | OMIM | 66.80 | 62.84 | 58.84 | 68.73 | 57.70 | 53.40 | 56.84 | **69.09** |
|  | Goh | **66.31** | 60.51 | 47.07 | 64.38 | 48.89 | 47.86 | 50.30 | 57.20 |
|  | Chen | 75.78 | 73.74 | 54.83 | **78.25** | 62.17 | 64.12 | 65.69 | 74.95 |
|  | Overall | 68.76 | 65.03 | 53.59 | **69.27** | 55.99 | 53.55 | 56.42 | 66.70 |
| bPPI | OMIM | 69.78 | 65.20 | 68.69 | **74.69** | 61.32 | 61.06 | 58.54 | 69.47 |
|  | Goh | **69.73** | 61.63 | 56.75 | 69.47 | 56.58 | 54.08 | 50.72 | 56.96 |
|  | Chen | 77.82 | 73.28 | 65.94 | **81.07** | 66.89 | 69.95 | 66.79 | 73.19 |
|  | Overall | 71.39 | 66.25 | 64.52 | **74.54** | 60.99 | 60.68 | 57.89 | 66.28 |
| Weighted bPPI | OMIM | 73.78 | 72.03 | 68.69 | **76.51** | 62.48 | 61.73 | 58.09 | 69.71 |
|  | Goh | **72.09** | 66.92 | 59.81 | 71.97 | 59.37 | 56.01 | 51.02 | 57.46 |
|  | Chen | 83.07 | 80.69 | 69.16 | **84.30** | 69.29 | 72.09 | 66.40 | 73.44 |
|  | Overall | 74.67 | 71.45 | 66.09 | **76.51** | 62.83 | 62.08 | 57.70 | 66.53 |

* Rows indicate the type of network and data set of seeds. An additional row is included for each network with the average results obtained with the seeds of all data sets (indicated as Overall). Highest values of AUC of each row are indicated in bold to highlight the best method.
