# Supplementary material for: Exploiting Protein-Protein Interaction Networks for Genome-Wide Disease-Gene Prioritization
Source: PLoS One. 2012 Sep 21;7(9):e43557. doi: 10.1371/journal.pone.0043557 (PMC3448640; doi:10.1371/journal.pone.0043557)
Supplement: Table S2 — P-values associated with the paired Wilcoxon signed rank test between Network Propagation and our two best prioritization methods on each data set using average AUCs over all networks. (DOC) [file pone.0043557.s006.doc]

**Table S2.**P-values associated with the paired Wilcoxon signed rank test between Network Propagation and our two best prioritization methods on each data set using average AUCs over all networks*

|  | NetCombo | NetScore |
| --- | --- | --- |
| OMIM | 2.00e-6 | 0.12 |
| Goh | 4.77e-7 | 4.77e-7 |
| Chen | 5.72e-6 | 8.20e-5 |

* The null hypothesis is that the algorithm we implemented (either NetCombo or NetScore) does not produce higher AUC values than Network Propagation algorithm.
