# Supplementary material for: Exploiting Protein-Protein Interaction Networks for Genome-Wide Disease-Gene Prioritization
Source: PLoS One. 2012 Sep 21;7(9):e43557. doi: 10.1371/journal.pone.0043557 (PMC3448640; doi:10.1371/journal.pone.0043557)
Supplement: Table S5 — Five-fold AUC (%) for each method averaged over all diseases within the data set and all interaction networks considering all non-seeds (genes not associated with the diseases) as negatives. (DOC) [file pone.0043557.s009.doc]

**Table S5.**Five-fold AUC (%) for each method averaged over all diseases within the data set and all interaction networks considering all non-seeds (genes not associated with the diseases) as negatives

| Data Set | NetScore | NetZcore | NetShort | NetCombo | Func. Flow | PageRank | Random Walk | Network Prop. |
| --- | --- | --- | --- | --- | --- | --- | --- | --- |
| OMIM | 75.50 | 73.11 | 69.19 | 74.57 | 72.67 | 70.70 | 70.82 | 72.51 |
| Goh | 73.64 | 70.84 | 66.27 | 73.05 | 68.00 | 68.62 | 69.09 | 70.56 |
| Chen | 81.27 | 78.75 | 68.66 | 80.59 | 74.88 | 76.14 | 75.81 | 78.14 |
