# Supplementary material for: Exploiting Protein-Protein Interaction Networks for Genome-Wide Disease-Gene Prioritization
Source: PLoS One. 2012 Sep 21;7(9):e43557. doi: 10.1371/journal.pone.0043557 (PMC3448640; doi:10.1371/journal.pone.0043557)
Supplement: Table S6 — The average NetCombo scores (the standard deviation is given in parenthesis) of CTD direct/indirect disease-genes and the genes with no-association in CTD and the p-value associated with the difference between these groups. (DOC) [file pone.0043557.s010.doc]

**Supplementary Table 6.** The average NetCombo scores (the standard deviation is given in parenthesis) of CTD direct/indirect disease-genes and the genes with no-association in CTD and the p-value associated with the difference between these groups

|  | Average score of direct association group | Average score of indirect association group | Average score of no-association group | P-value of direct vs indirect  (*P ≤ p)* | P-value of direct vs no-association  (*P ≤ p)* |
| --- | --- | --- | --- | --- | --- |
| AD | 0.134 (0.093) | 0.069 (0.046) | 0.054 (0.038) | 5.8e-5 | 2.6e-6 |
| Diabetes | 0.089 (0.085) | 0.058 (0.035) | 0.049 (0.028) | 1.3e-3 | 5.2e-5 |
| AIDS | 0.123 (0.075) | 0.079 (0.032) | 0.071 (0.029) | 1.3e-3 | 2.6e-4 |
