# Supplementary material for: Exploiting Protein-Protein Interaction Networks for Genome-Wide Disease-Gene Prioritization
Source: PLoS One. 2012 Sep 21;7(9):e43557. doi: 10.1371/journal.pone.0043557 (PMC3448640; doi:10.1371/journal.pone.0043557)
Supplement: Table S9 — Number and size of the connected components other than the largest connected component (LCC) in the network. (DOC) [file pone.0043557.s013.doc]

**Table S9.** Number and size of the connected components other than the largest connected component (LCC) in the network

|  | **Number of components with size k in the network** | | | |
| --- | --- | --- | --- | --- |
| **size of the component (k)** | Goh | Entrez | PPI | bPPI |
| 2 | 92 | 70 | 49 | 68 |
| 3 | 16 | 13 | 17 | 18 |
| 4 | 3 | 6 | 5 | 7 |
| 5 | 2 | 1 | 2 | 2 |
| 6 | 0 | 0 | 1 | 1 |
