# Supplementary material for: Exploiting Protein-Protein Interaction Networks for Genome-Wide Disease-Gene Prioritization
Source: PLoS One. 2012 Sep 21;7(9):e43557. doi: 10.1371/journal.pone.0043557 (PMC3448640; doi:10.1371/journal.pone.0043557)
Supplement: Table S10 — Interaction data sets used in the analysis. (DOC) [file pone.0043557.s014.doc]

**Table S10.**Interaction data sets used in the analysis*

| Network | Sources | Date of retrieval or version | # of nodes in LCC | # of edges in LCC |
| --- | --- | --- | --- | --- |
| Goh [19] | Interactions collected from literature (i.e. large-scale Y2H experiments by Rual et al. [20] and Stelzl et al. [21]) | n/a | 7279 | 21911 |
| Entrez | Interactions collected from BIND [22], HPRD [9] | January 2010 | 10119 | 49826 |
| PPI network (BIANA [1]) | DIP [8] | January 2009 | 12506 | 205966 |
|  | HPRD [9] | September 2007 |  |  |
|  | IntAct [10] | January 2009 |  |  |
|  | MIPS (MPACT) [11,23] | October 2008 |  |  |
|  | BioGRID [12] | 2.0.49 |  |  |
| bPPI network | Same interaction sources as PPI network above, but interactions detected by TAP are excluded. | Same as above | 11250 | 59220 |
| weighted bPPI network | Same interactions as bPPI network above, but interactions are weighted by corresponding scores in STRING database (if exist). | Same as above | 11250 | 59220 |

* See Methods S1 for the references.
