# Supplementary material for: Exploiting Protein-Protein Interaction Networks for Genome-Wide Disease-Gene Prioritization
Source: PLoS One. 2012 Sep 21;7(9):e43557. doi: 10.1371/journal.pone.0043557 (PMC3448640; doi:10.1371/journal.pone.0043557)
Supplement: Table S11 — Number of disease-gene associations covered in each network. (DOC) [file pone.0043557.s015.doc]

**Table S11.**Nubmer of disease-gene associations covered in each network

|  |  | **# of genes** | **# of proteins covered by the largest connected component (LCC) of the interaction network** | | | | |
| --- | --- | --- | --- | --- | --- | --- | --- |
| **Data set** | **Phenotype** |  | **Goh** | **Entrez** | | **PPI** | **bPPI** |
|  | adrenoleukodystrophy | 13 | 6 | 7 | | 7 | 7 |
|  | aids | 17 | 7 | 9 | | 7 | 7 |
|  | alzheimer | 53 | 17 | 18 | | 18 | 18 |
|  | amyloidosis | 13 | 6 | 7 | | 9 | 9 |
|  | amyotrophic | 49 | 15 | 18 | | 20 | 19 |
|  | anemia | 37 | 10 | 13 | | 13 | 11 |
|  | arrhythmogenic | 29 | 10 | 10 | | 10 | 10 |
|  | asthma | 38 | 12 | 14 | | 16 | 16 |
|  | atrial | 63 | 13 | 14 | | 19 | 18 |
|  | autism | 51 | 10 | 12 | | 12 | 12 |
|  | autoimmune | 37 | 9 | 10 | | 15 | 14 |
|  | bardet biedl | 43 | 9 | 12 | | 17 | 16 |
|  | blood | 80 | 33 | 36 | | 43 | 43 |
|  | brachydactyly | 25 | 8 | 9 | | 9 | 9 |
|  | breast | 61 | 27 | 29 | | 34 | 32 |
|  | cardiomyopathy | 167 | 43 | 52 | | 60 | 55 |
|  | cataract | 80 | 23 | 25 | | 27 | 27 |
|  | cerebral | 40 | 11 | 16 | | 20 | 18 |
|  | charcot marie tooth | 104 | 24 | 34 | | 39 | 37 |
|  | colon | 18 | 8 | 8 | | 10 | 10 |
|  | colorectal | 92 | 31 | 36 | | 39 | 38 |
|  | combined | 41 | 15 | 18 | | 25 | 23 |
|  | cone rod | 73 | 14 | 19 | | 24 | 24 |
|  | congenital | 96 | 17 | 25 | | 29 | 26 |
|  | coronary | 47 | 16 | 19 | | 19 | 18 |
|  | deafness | 261 | 43 | 51 | | 53 | 52 |
|  | dementia | 24 | 9 | 10 | | 11 | 11 |
|  | diabetes | 152 | 50 | 57 | | 60 | 56 |
|  | diamond blackfan | 22 | 7 | 7 | | 11 | 9 |
|  | dystonia | 20 | 11 | 13 | | 15 | 15 |
|  | ectodermal | 42 | 9 | 12 | | 13 | 13 |
|  | epidermolysis | 24 | 13 | 14 | | 17 | 17 |
|  | epilepsy | 115 | 19 | 26 | | 26 | 24 |
|  | epileptic | 52 | 11 | 12 | | 15 | 13 |
|  | epiphyseal | 23 | 9 | 9 | | 9 | 9 |
|  | esophageal | 22 | 12 | 14 | | 15 | 15 |
|  | factor | 12 | 9 | 9 | | 16 | 15 |
|  | fanconi | 54 | 15 | 21 | | 22 | 22 |
|  | gastric | 36 | 18 | 18 | | 25 | 24 |
|  | glomerulosclerosis | 16 | 6 | 6 | | 7 | 7 |
|  | glycogen | 38 | 11 | 16 | | 14 | 14 |
|  | hemolytic | 43 | 18 | 22 | | 27 | 25 |
|  | hemophagocytic | 18 | 8 | 8 | | 8 | 8 |
|  | hepatocellular | 17 | 7 | 8 | | 8 | 8 |
|  | high | 26 | 9 | 9 | | 10 | 10 |
|  | hypercholesterolemia | 23 | 10 | 13 | | 15 | 14 |
| OMIM | hypertension | 35 | 10 | 11 | | 13 | 13 |
|  | ichthyosis | 51 | 13 | 17 | | 16 | 14 |
|  | immunodeficiency | 49 | 21 | 26 | | 27 | 27 |
|  | keratosis | 20 | 6 | 9 | | 10 | 10 |
|  | leigh | 36 | 8 | 9 | | 15 | 14 |
|  | leukemia | 125 | 58 | 63 | | 80 | 77 |
|  | long | 47 | 13 | 14 | | 16 | 16 |
|  | lung | 36 | 13 | 15 | | 19 | 18 |
|  | lymphoma | 24 | 15 | 15 | | 16 | 16 |
|  | macular | 93 | 23 | 25 | | 30 | 29 |
|  | malaria | 33 | 15 | 15 | | 20 | 20 |
|  | melanoma | 30 | 9 | 11 | | 10 | 10 |
|  | mental | 284 | 55 | 71 | | 75 | 71 |
|  | microcephaly | 33 | 7 | 8 | | 11 | 10 |
|  | microphthalmia | 57 | 11 | 13 | | 13 | 13 |
|  | microvascular | 23 | 11 | 11 | | 11 | 11 |
|  | mitochondrial | 92 | 18 | 24 | | 23 | 20 |
|  | multiple | 55 | 19 | 20 | | 24 | 23 |
|  | muscular | 105 | 17 | 18 | | 21 | 19 |
|  | myasthenic | 19 | 8 | 9 | | 9 | 9 |
|  | myocardial | 35 | 12 | 13 | | 15 | 14 |
|  | myopathy | 76 | 20 | 25 | | 27 | 25 |
|  | neuropathy | 74 | 16 | 22 | | 27 | 22 |
|  | noonan | 28 | 13 | 14 | | 15 | 15 |
|  | obesity | 57 | 22 | 24 | | 27 | 25 |
|  | osteopetrosis | 33 | 7 | 8 | | 8 | 8 |
|  | ovarian | 41 | 15 | 17 | | 24 | 24 |
|  | pancreatic | 42 | 16 | 17 | | 18 | 18 |
|  | parkinson | 60 | 12 | 12 | | 18 | 15 |
|  | pituitary | 23 | 10 | 12 | | 12 | 11 |
|  | prostate | 74 | 29 | 29 | | 32 | 32 |
|  | pulmonary | 29 | 12 | 16 | | 17 | 17 |
|  | renal | 61 | 19 | 24 | | 25 | 24 |
|  | retinitis | 168 | 42 | 49 | | 63 | 60 |
|  | rheumatoid | 27 | 16 | 19 | | 24 | 21 |
|  | schizophrenia | 34 | 10 | 12 | | 15 | 14 |
|  | severe | 23 | 11 | 14 | | 15 | 14 |
|  | short | 38 | 8 | 11 | | 12 | 11 |
|  | spastic | 91 | 15 | 19 | | 23 | 20 |
|  | spinocerebellar | 97 | 18 | 24 | | 35 | 33 |
|  | systemic | 50 | 16 | 18 | | 21 | 21 |
|  | thrombophilia | 19 | 17 | 19 | | 20 | 20 |
|  | thyroid | 65 | 24 | 29 | | 30 | 29 |
|  | usher | 33 | 6 | 6 | | 6 | 6 |
|  | xeroderma | 19 | 9 | 10 | | 12 | 11 |
|  | zellweger | 24 | 11 | 12 | | 16 | 16 |
|  | bone | 119 | 35 | 42 | | 42 | 42 |
|  | cancer | 460 | 224 | 252 | | 299 | 283 |
|  | cardiovascular | 243 | 101 | 115 | | 130 | 121 |
|  | connective tissue | 128 | 52 | 64 | | 83 | 67 |
|  | dermatological | 190 | 78 | 90 | | 105 | 92 |
|  | developmental | 126 | 54 | 60 | | 73 | 66 |
|  | ear,nose,throat | 125 | 29 | 38 | | 36 | 36 |
|  | endocrine | 202 | 98 | 111 | | 121 | 119 |
|  | gastrointestinal | 78 | 23 | 27 | | 28 | 25 |
|  | hematological | 282 | 145 | 169 | | 200 | 195 |
| Goh | immunological | 250 | 124 | 138 | | 171 | 155 |
|  | metabolic | 526 | 165 | 237 | | 282 | 241 |
|  | multiple | 550 | 196 | 233 | | 267 | 252 |
|  | muscular | 173 | 59 | 69 | | 76 | 71 |
|  | neurological | 626 | 214 | 262 | | 310 | 292 |
|  | nutritional | 43 | 21 | 23 | | 25 | 23 |
|  | ophthamological | 292 | 100 | 117 | | 146 | 140 |
|  | psychiatric | 65 | 24 | 28 | | 32 | 30 |
|  | renal | 144 | 48 | 65 | | 76 | 72 |
|  | respiratory | 69 | 28 | 36 | | 42 | 39 |
|  | skeletal | 164 | 58 | 66 | | 66 | 65 |
|  | atherosclerosis | 35 | 30 | 33 | | 38 | 38 |
|  | autism | 41 | 25 | 34 | | 43 | 39 |
|  | cervical carcinoma | 38 | 31 | 36 | | 61 | 51 |
|  | cirrhosis | 30 | 23 | 25 | | 34 | 33 |
|  | endometrial carcinoma | 33 | 34 | 35 | | 40 | 39 |
|  | endometriosis | 43 | 39 | 43 | | 73 | 56 |
|  | epilepsy | 36 | 26 | 36 | | 44 | 41 |
|  | graves disease | 36 | 35 | 36 | | 60 | 50 |
|  | hypercholesterolaemia | 32 | 19 | 25 | | 29 | 26 |
| Chen | inflammatory bowel disease | 36 | 32 | 32 | | 45 | 44 |
|  | ischaemic stroke | 44 | 45 | 47 | | 74 | 64 |
|  | lymphoma | 42 | 41 | 44 | | 79 | 57 |
|  | migraine | 33 | 26 | 31 | | 36 | 33 |
|  | myocardial ischemia | 39 | 36 | 38 | | 43 | 43 |
|  | neural tube defects | 38 | 21 | 24 | | 28 | 24 |
|  | osteoarthritis | 41 | 37 | 40 | | 51 | 49 |
|  | pancreatitis | 31 | 26 | 26 | | 53 | 37 |
|  | systemic scleroderma | 31 | 29 | 30 | 47 | | 37 |
|  | ulcerative colitis | 34 | 33 | 36 | 49 | | 47 |
