# Supplementary material for: Exploiting Protein-Protein Interaction Networks for Genome-Wide Disease-Gene Prioritization
Source: PLoS One. 2012 Sep 21;7(9):e43557. doi: 10.1371/journal.pone.0043557 (PMC3448640; doi:10.1371/journal.pone.0043557)
Supplement: Table S12 — Genes used for parameter optimization. (DOC) [file pone.0043557.s016.doc]

**Table S12.**Genes used for parameter optimization

| Phenotype | Gene Symbols |
| --- | --- |
| Aneurysm | ELN, PKD1, NOS3, COL3A1, APOE, PKD2, COL1A2, MMP9, SERPINA1, TIMP1, MMP2, ACE, ENG, TIMP2, LOX, NOS2, MFAP4, COL4A1, CST3, SPARCL1, VCAM1, COL6A2, TIMP3, TGFBRE, SERPINE1, FBN2, LIMK1, ANIB4, HMOX1, ACE2, VCAN, TSPAN5, MPRIP, LOXL3, LOXL4, LTBP4, COPS3, AKAP10, FGF12, LPA, MUC17, SPARC, GRIN3B, COL1A1, FGF2, GRAP, LOXL2, ECE2, TGFBR2, OFD1, HSPG2, GSTO1, RAI1, CXCL14, CCL2, PAQR7, SEPT4, GDF6, PDK2, SOX17, COL4A4, KLK8, S100A11, MTMR11, F5, IGFBP7, CTSB, MMP3, TNFRSF13B, HPN, ABCC5, FN1, LOXL1, PLCD1, PRODH, TRPC4, MAPK7, MTRR, HP, ACVR1, COL4A5, TGFBI, COL4A3, RASA1, PRG2, TGFBR3, ACVRL1, SLC19A1, COL6A1, FGD1, TCN2, ITGB4, CYBA, VLDLR, MAP3K14, TRPC1, MMP12, PRKD1, PPM2C, CTSS, GAA, FBN1, ABCC2, ABCB4, ECE1, KEL, FLNA, CTSK, C2, HMBS, CBS, DLL1, FOLR1, MTR, MMP7, APOB, IL1B, FOXD3, JAG1, APOC3, TGFBR1, EDNRB, CTSG, SREBF1, DARC, PDGFB, CFB, C4B, SERPINA3, IGFBP2, VHL, ANGPT1, ADM, TGFB1, SLC6A3, FGF1, F12, CASP1, RELA, EDN1, CD68, MGAM, MTHFR, APP, SPP1, MMP1, NFKB1, CDKN2A, DHFR, HIF1A, APC, TGFA, NOS1, GAPDH, CST6, CD44, IL10, NPR1, B2M, VEGFA, C3, PCNA, AGT, VWF, SERPINC1, TNF, IL6, KNG1 |
| Breast Cancer | BRCA1, ERBB2, BRCA2, ESR1, TP53, PGR, EGFR, CCND1, ABCG2, MYC, ESR2, AKT1, CYP19A1, CDH1, BCL2, CDKN1A, NCOA3, HRAS, CHEK2, ATM, PIK3CG, EGF, MUC1, ERBB3, PTEN, RB1, MKI67, CDKN1B, CYP1B1, KRT19, NRG1, SCGB2A2, CTSD, CYP1A1, AR, TOP2A, VEGFA, RASSF1, TGFA, ERBB4, SERPINB5, CTNNB1, NME1, IGF1R, FGF3, BAX, BARD1, CDKN2A, TFF1, MDM2, JUN, RAD51, INTS2, RARB, TGFB1, CYP17A1, MLH1, GSTP1, HEATR6, ABCC1, AHR, CDK2, SP1, GSTM1, HIF1A, NCOA1, ABCB1, PIK3CA, IGF1, MMP9, XRCC1, TERT, COMT, FHIT, SCGB1A1, MMP2, MSH2, MAPK3, SRC, TSG101, PALB2, AURKA, NFKB1, GSTT1, CDK4, XRCC3, RARA, BRMS1, PLAU, ABCC3, KRT5, SULT1A1, TNFSF10, IGF2, AREG, VDR, ZNF451, STAT3, PLAUR, FGFR2, TGFBR2, SNCG, STK11, ERCC2, SFN, CCNA2, PARP1, ABCC2, MMP11, BRIP1, FRAP1, PTGS2, FANCD2, CASP8, MTA1, PRLR, KLK10, CXCR4, ETV4, CD44, KRAS, IGFBP3, WNT1, APC, HDAC1, PTHLH, CASP3, GRB7, BCAS1, CXCL12, CAV1, TGFBR1, CYP2D6, S100A4, NCOR1, WWOX, RHOC, CCND2, GADD45A, SLC5A5, BCL2L1, HSD17B1, CTTN, EPCAM, CCNB1, TFAP2C, IRS1, DNMT1, NCOR2, PTK2, WT1, ETS1, ELF3, MTHFR, MIR21, MMP1, PIP, PHB2, FOXA1, XRCC2, SMAD4, NAT2, NCOA2, ID1, MAPK8, PHB, MAGED2, SPDEF, SCGB3A1, TP73, SOD2, ZNF217, FASN, SPP1, CASP9, ABCC5, YBX1, MAPK14, MVP, KLK5, DIRAS3, EZH2, AKT2, PPM1D, STS, SLC45A2, CDK6, LASS2, MMP14, CDC25A, CASP7, TFAP2A, MUCL1, FGFR1, HIC1, CD82, ETV1, GPER, WISP2, CCNE1, BCAR1, BCAR3, TOX3, ABCC4, KLK6, DEPDC1B, FGFR4, CYR61, ACTB, C11orf30, ERCC1, KDR, FGF4, SHC1, RAD50, ESRRA, CYP24A1, HOXB13, ANKRD30A, KRT8, SFRP1, MRE11A, RPL10, PELP1, TYMS, CDH13, KLK14, TACC1, SLC39A6, LASP1, FOS, STAT5A, NAT1, HOXA5, MIB1, RAF1, TIMP3, E2F1, ETS2, PAK1, RARG, FANCC, SNAI2, VEGFC, NARG2, WNT5A, MAP2K1, PLK1, SKP2, BMI1, GSK3B, PPARG, CD9, CREBBP, BECN1, NBR2, FANCA, IL17RB, DLC1, BAG1, NCOA6, ACSBG1, MTA3, RBBP8, KRT18, MSH6, S100A7, MAP2K4, TWIST1, TDGF1, TIMP2, MAP3K1, CTCF, BLID, PDCD4, FOXO3, RHOBTB2, IGFBP7, CDH3, WNT10B, CYP3A5, ARNT, IGF2R, UGT1A1, TRIM25, CHUK, SULT1E1, FIGF, CCND3, SMARCA4, AKT3, KRT14, TPD52, KIAA1967, RAC1, MET, CTNNA1, JARID1B, RNF11, WISP3, XPC, EPHA2, SAGE1, CHEK1, GATA3, IGFBP5, ARL11, STAT5B, ZNF350, EIF4E, E2F4, RPS6KB1, TBX2, IRS2, ERCC4, CTGF, CTAG1B, CTAG1A, NBR1, SERPINE1, GRB2, SLIT2, MACROD1, BRCA1P1, CSF1R, KLK13, C11orf17, RAD52, ZEB1, BUB1B, HMGA1, WNT3, ZNF703, IL24, KISS1, FANCG, ODZ4, PRDM2, ST14, NRIP1, SMAD3, XRCC4, CCL16, PTK6, MMP3, RELA, HGF, NQO1, MYB, KAT2B, SSX2, CLU, NBN, GDF15, CYP3A4, ELAVL1, SMARCA1, BSG, FLT1, CCNE2, FOSL1, CSN2, XIAP, RBBP7, NR2F1, HDAC6, CUL4A, CDKN2B, CA9, HSD17B2, SYK, ZNF35, IGFBP4, ATPBD3, TGFB2, BIK, MYBL2, KLK4, TIMP1, BIRC5, STARD3, HSD17BP1, HYAL2, SRD5A2, BAK1, WNT7B, TFF2, LSP1, ZEB2, SLCO2B1, OSM, TYMP, ABCB4, SAFB, BCL2L11, AKR1B1, RHOA, PLEKHB1, ODZ3, MIR10B, FGR, NRP1, SHFM1, DNMT3B, FANCF, WNT2, SIRT1, STAT1, DPYD, CBFA2T3, MMP13, SIPA1, NR1I2, XBP1, ACACA, CASP10, SSTR2, CEBPD, HSD17B13, CEBPB, GHRHR, TP53BP1, ING1, SCGB1D2, TCF7L2, CREB1, HSD17B7, CDK10, PHIP, TNFRSF10B, PTTG1, MAD2L1, PIN1, SMYD3, VHL, TFF3, SEMA3B, CYP27B1, BIN1, CUX1, FBLN1, NDRG1, PMS2, OSGIN1, CDH11, REL, PTPRG, EBAG9, KRT17, STC2, CASP6, ETV2, THBS1, RB1CC1, DLX4, PPP2R1B, MYCN, WNT9A, GPX1, SNAI1, FGF7, SP3, PXN, CYP1A2, TP63, PRKCA, LZTS1, HOXB7, PPM2C, BCAS3, RBP1, EP300, ITIH5, NRG3, LEF1, BCL2L12, ID2, FANCL, MED1, NOV, BLM, BBC3, LCP1, MTA2, BANP, PTN, CTNND1, PRKDC, DKK1, MT1E, STMN1, RUNX3, CLDN4, DAPK1, XRCC6, MMP7, KLK2, UGT2B15, S100P, RBM45, DPH1, INTS6, SETMAR, SMAD7, RAB6C, SMAD2, SLCO1B3, ARHGAP1, FRK, LGALS3, STC1, SPARC, SAFB2, HBEGF, TGFBR3, IGFBP2, RAD51C, CARM1, UHRF1, HELZ, RRM2, WNT2B, NOTCH1, FABP3, TOP2B, ABCF2, DCC, CACNA2D2, CUL1, TOPBP1, SEPT9, PRIMA1, EPB41L3, PCNA, EIF3H, GRB14, CDC2, CYP2B6, EGR1, MUC16, UBE2C, NRG2, NPM1, RELB, CLCA2, CISH, SCGB2A1, SLC19A1, CDH2, POU4F2, OGG1, TGFB3, DDIT3, CST6, IRF1, INTS5, RERG, EIF4EBP1, NPY6R, ABCC11, UIMC1, ID4, AXIN1, HDAC2, WNT5B, IBSP, DUSP1, CSK, PPID, CASC3, SMARCE1, MT1H, ZFP36L1, SMURF2, KLK9, ITGA6, MIRHG2, COPS5, NME2, UPP1, FANCE, NOTCH4, SLCO1B1, BUB1, TNS1, PIK3CB, EMP3, WRN, NUPR1, ROBO1, TUSC4, TACC2, MAGEA3, MAPK1, LYPD5, MIR206, H19, WNT10A, LIMA1, MST1, FAP, WWP1, ODZ2, TSP50, JAK2, NRG4, GAB2, SLC22A7, LOXL2, KIF14, GLI1, AKAP1, GNRHR, MLLT11, GABRP, RNF115, NR5A2, WNT9B, PHLDA1, H2AFX, PTCH1, RAD51L3, UGCG, MAPK7, CAV2, FGF1, FBXW7, JUP, PRC1, BTG2, USF2, POSTN, RPL19, ENPP2, MT1X, MMP17, SOX4, TRIM24, LATS2, INTS3, C3orf35, IKBKE, SPINT1, BMPR1B, BAD, PUM1, NR2F6, FGFBP1, APOD, KLK7, NCOA4, EPAS1, FGF8, CTBP1, CTDSPL, ITGB3BP, BRAP, WIF1, TNFSF11, CCL27, GSTM3, ABCB11, WASF3, MIR205, MTDH, KLF4, LIG4, CDC25C, TNFRSF10C, BAGE, AKR1C3, PTPRJ, SFRP5, DAP, TGM2, TTC4, C17orf37, LYVE1, FBXW10, PRDX2, TIMP4, PA2G4, MMP15, USF1, SEMA3F, EIF3E, MCTS1, BMPR1A, FAM175A, PERLD1, CCAR1, KLK8, HAS2, DDB2, ADAM9, SP4, DFNA5, MIR335, BUB3, SULF2, PMS1, PLXNB1, AXIN2, ITGB4, MDC1, ANXA5, UGT2B7, C1orf38, TPD52L1, LMO4, MRPL19, CBR3, GAPDH, CCNA1, HTATIP2, NFYA, MTSS1, WISP1, ERLIN2, MIR27A, GRN, GPC3, PDS5B, RASSF5, SUZ12, CDH5, SRA1, TNFRSF10A, RECK, WNT6, DDIT4, ERCC5, CIZ1, PSMD7, MIR373, IKBKB, PRKCD, NUP88, SLC35A2, GSN, KLK15, PTPN13, PTPRT, MAD1L1, NAP1L4, TSC2, NR1D1, MUC4, EXTL1, CLDN1, ARID4A, MT1F, CDKN2C, RORA, FOSL2, CEACAM5, DAB2, ADH1C, NEK2, RAD51L1, ODZ1, BCAR4, DDHD2, SULT1A2, GMNN, DNMT3A, RRM1, LIMK1, RAD54B, HDAC3, DCD, ID3, CCNG2, CSAG3, CLDN7, RXRA, RBM5, EFNA1, CYP2C19, YY1, CEP55, LOXL3, CAGE1, DICER1, BHLHE40, HSP90AA2, SIAH1, ERCC6, NEDD9, SULF1, NR3C1, RNF41, BNIP3, HSP90AB1, ICAM5, LRIG1, SFRP2, MIR145, LOXL4, MMP12, FOXP1, RNF31, DCTPP1, SOCS2, TACSTD2, CDC25B, MTUS1, CASP4, AHRR, AMACR, TBX3, LEPR, JUND, MT2A, CCNC, SIX1, KLK11, CXCR7, BCAS2, LCN2, TXNIP, PRMT1, SLC30A1, ERBB2IP, RAB11FIP1, SLC19A3, FANCI, AXL, KLF5, NRP2, S100A2, HOXD10, CEACAM1, MIA, FGF6, ECM1, MCPH1, HMMR, ANP32A, MMP27, GSDMA, MIR152, FBXO31, MIR663, SASH1, ZBTB10, PBOV1, DVL2, GLI2, XPA, TERC, ARHGAP5, SLCO4A1, SCAF1, MIR210, IL32, HOXC6, IGF2BP1, GPX4, TOB1, SULT2B1, NPTN, UXT, FHL2, CRABP2, FLT4, THRSP, ADIPOR1, EVL, CASP14, LOX, SLC2A12, PPP1R13L, POTED, MED23, FOXM1, PTP4A1, IER3, TXNRD1, NDC80, CRKRS, SEC14L1, ANO4, APAF1, LIG3, HOXA1, BCL3, SEL1L, BNIP3L, TP53INP2, FOXC2, ILK, GABPA, GSTO1, DNAJC6, ADAM17, CDKN1C, BIN2, CXCL14, AKR1C1, RPRM, APEX1, TUBB1, SOCS3, PITX2, FKBP4, SLC22A18AS, TSPAN13, CLDN3, SPRY1, RBM10, PLD2, GPAA1, MIR126, C21orf7, TMPRSS6, MMP10, FGF10, BIRC3, NFE2L2, SDHC, MAP2K5, MMP28, AMHR2, PPP2R5B, RRP1B, MIR9-1, PIBF1, NHLH1, ADAM12, CEACAM6, BMP6, KLK1, WNT4, SMARCB1, TAF2, E4F1, KIF22, ZNF410, EGLN3, AKAP13, FOXO1, ADAM15, CHFR, ELF5, MIR146A, CFLAR, ALCAM, TPD52L2, ARHGAP9, IMMP2L, PTENP1, STARD8, MUT, RAB25, ATP1B2, PTP4A3, DMBT1, CSNK2A1, EPHB6, ICAM4, ST13, WNT3A, UBE2B, ITGB3, ITGB5, PDZK1, RNASEL, LPAR2, PIGK, ARHGAP29, TMEM97, FAT4, PNCK, MT3, CUL3, E2F5, PLAGL1, HSP90B1, EEF1A2, PPARD, SMARCA2, GRB10, RIN1, TSPAN8, NPAT, CHN2, INSC, ETV6, BRD4, CSE1L, ARHGAP20, SRGAP1, TSKU, VWA5A, ANO3, CLPTM1, SUV420H2, SPOCK2, MIR98, FOXE1, CIB1, TXN2, ESRRG, GPNMB, CLDN6, DVL3, MUC2, WNT11, MMRN1, TTK, ZFHX3, CYP3A7, CCNH, MT1A, ARHGAP8, BRI3BP, TMSB15A, TMSB15B, ADAMTS15, KLK12, YWHAZ, DFFA, IL11, IKBKG, CENPF, SET, TNFRSF11B, TRAF4, DACH1, TUSC3, DKK3, MEN1, SIM2, MRPS30, GSDMB, ORAOV1, TNS4, CTHRC1, SLC16A3, SRGAP2, PHLPPL, RBL2, CHI3L1, FABP5, RPS6KA5, TUBB3, GSTA1, PFN1, FOLH1, ANXA1, REEP5, TFAP2B, RBM9, MT1G, KRT20, E2F3, LY6K, DIXDC1, LASS6, TRIM16, CYB561D2, CSTF1, FOXF1, NUMA1, LMO1, LSM1, FZD6, CD63, MMP16, UBE3A, E2F6, SLC22A18, KCNH8, PDGFRL, C6orf66, RTEL1, GALNT1, HECTD1, CD3EAP, POLD1, SOX18, FGF20, TK1, ARF6, ERP29, PRDM1, TXNRD2, SLC22A8, REXO4, IFI35, ABCC12, CCNDBP1, CEBPZ, TERF1, ARHGDIB, RRAS2, MED14, FOSB, MST1R, CALCR, SUV39H1, DSC3, HEYL, UBE2S, PRICKLE2, HMG20B, HYAL3, LIMD1, MECP2, RHOB, CITED1, CHKA, TRRAP, MTR, C16orf53, BARX2, DAB2IP, BRCC3, SEMA6B, DAAM2, KLF12, TNKS, TACC3, VTCN1, PMAIP1, THRB, UBR5, CDKN2D, HGFAC, SIK1, WHSC1L1, NUMB, LMO7, ST7, HS3ST2, CBX5, CYP26A1, MMP26, MAP3K7, HSF1, MAD2L2, MFGE8, MIR141, NEK3, CUL4B, RNF6, NAP1L1, MAGEC1, TSC22D1, KISS1R, EPHB4, RAD21, SULT2A1, PSMC4, BOK, DNAJB7, CCNB2, TXNDC12, VPS52, GDE1, NPAS2, NOTCH2, HMGA2, HSPA8, AURKB, SMURF1, ELAC2, MSX2, PPP2R1A, CDC42SE2, QSOX1, ARHGAP4, MIR31, AKAP12, MCL1, ITGA3, MAPK9, TJP1, SPINT2, ANAPC1, CRTAC1, DUSP22, PAK6, RAD54L, APC2, TNFRSF11A, ADH1B, HSPA5, PIAS3, LUM, TMEM115, ASAP1, NKD2, RPS6KA6, LPAR1, NR2F2, SLC22A1, HRK, UBE2I, MDK, PPP1R15A, XRCC5, ADRM1, WNT8A, FRS3, NPNT, TNKS2, TAX1BP1, ARID1A, BTG1, FFAR1, NEK10, C16orf3, FBXL8, NOP16, USP49, RAB9B, CRIPAK, KIAA1244, SCYL3, PHBP1, RP13-36C9.1, RNF152, TMCO4, CEACAM19, VSIG1, C17orf28, KIAA0284, FAM19A4, SMYD4, MIR9-3, RASSF3, GPATCH2, SLTM, PCDHGB6, USP35, TTC39A, IGFL1P2, IGFL1P1, TSSC2, CPNE7, SNORA74A, KIAA1632, MIR661, SHCL1, OR10J3, CWF19L2, C3orf16, IGFL4, PRICKLE4, ANKRD30B, C19orf62, PROX1, GGPS1, DDB1, CYP2C18, TNS3, ANO2, IFI6, LATS1, MAP2K6, EREG, PABPC1, GIPC1, ISG15, PEBP1, CASR, NMI, THOC1, ST18, CDC20, AKR1C2, MCM2, RAD9A, BCL2A1, MN1, MUTYH, SRGAP3, FGF16, NR1I3, SATB1, TRPS1, CASP2, CSTA, ANO1, EBNA1BP2, CELSR2, NFATC4, CITED2, MMP19, RPS6KA1, RPS9, SDC2, MTAP, MGMT, NFKB2, MIR17, MT1B, PDSS2, AMOT, STMN3, C8orf4, GSPT1, EIF2C2, SYCP1, MYCL1, PRDX1, EZR, PSPC1, SLC25A5, E2F2, ENOX2, IQWD1, PRICKLE1, STEAP1, FANCM, GSTM4, PEA15, RICTOR, PBK, ARID4B, MSH3, PPIA, UVRAG, PIGX, ITGB8, EED, GADD45B, EHF, SLC10A1, MTRR, CDC42, ZYX, RBM3, RNF8, DAAM1, SH3RF1, PTPRF, PSEN2, UGT1A10, RBBP4, PHLDA2, SLC39A7, RNF4, FOXL1, CCNG1, SERPINB2, CUL5, VAV2, TCAP, SLC9A3R1, LRRC15, TINAGL1, TM4SF1, UGT8, CDV3, CCT5, ISG20, ZNF398, MIR125A, POTEH, MIR421, FAM19A1, C1orf152, MT1JP, TSHZ2, CNTNAP4, KIAA1245, IFI27L1, MIR584, CTD-2267G17.3, XAGE2, CCDC11, YPEL2, TSSC1, HIST2H2AA4, HIST2H2AA3, TRAF2, TMEM25, ZNF276, ZNHIT6, FRMPD1, DNAH17, IGFL1, YWHAD, CMTM4, FAM110A, TTC9, HUNK, DDX54, TMEM14C, CUEDC2, MIR453, PPAPDC1A, FBXO10, LRRC29, TPD52L3, LETM2, ECHDC1, MIR200A, TBC1D5, HIST1H2AC, PLEKHG2, KIAA0100, THBS2, CDK7, ACVR1, KRT9, CPD, BAG4, ADAM28, PIK3R3, YEATS4, RSU1, SOX7, RFC4, MYST2, DIABLO, RPSA, SOCS1, CXCL1, RHOU, GPRC5A, HECW1, SRF, LILRB1, HPN, SIVA1, PPP1CB, PSMC3IP, RBMX, FRAT1, SMO, FZD1, CENPA, TYRP1, APOBEC3B, IGF2AS, TMPRSS2, TGFB1I1, POU2F1, SFRP4, CSNK1E, EGLN2, RPL13, STAMBP, TAF10, CDCP1, FRS2, MXI1, ALOX12, C3orf10, MELK, ING2, TNFRSF10D, HPSE, MAGEC2, POLL, RPL13A, SLC44A1, ST8SIA1, BIRC7, HIPK2, KANK2, S100A7A, TUSC1, IGFBPL1, ZPBP2, PBXIP1, INTS12, ARHGAP19, SUMO1P1, RUNDC3B, PPIAL4A, SERAC1, NXPH2, LRRC49, TDRD3, ARHGEF5, PPAPDC1B, KIAA1468, ARHGAP23, CMTM5, NANOGP8, ADAMTS17, IRX5, ZNF14, WTIP, FAM127A, MRPL32, BCORL1, HDAC7, LRRC26, CKAP5, TERF2, MGAT5, DHH, ATF4, AKR1CL2, ARL2, DUSP6, BTC, ATF3, PDGFRB, SSX4B, SSX4, ANGPTL4, KSR1, JAG1, UBE2N, RRAS, BCYRN1, ACVR2B, MAP3K3, CLDN5, PRKAR2B, MAP4, TPT1, BCCIP, TNFAIP8, MRC2, RFC2, FZD10, HNRNPK, TXN, HIST1H1E, SULT1B1, FGF17, CASD1, PAICS, RSF1, ITGA8, CCBE1, RNF146, KIAA1324, ZNF652, NFAM1, RASL10B, RBM35A, ADAMTSL3, THAP10, TTTY1, MIR497, EPSTI1, RP6-166C19.11, RP6-166C19.1, RP6-166C19.2, RP6-166C19.3, RP6-166C19.4, RP6-166C19.5, RP6-166C19.6, CT47.8, RP6-166C19.9, RP6-166C19.10, CT47.7, ARHGAP18, CPNE6, LEPREL2, FAM102A, CMTM3, ANO9, DNAJC12, SDF2L1, ANKRD26, TRIM49, PPP2R5A, ARHGAP10, SHISA2, KPNA5, OGDHL, ARHGAP15, MED30, SLC2A6, KPNA1, ITGA9, TRAF1, UBE2E3, TMSB10, HLTF, CKS2, TNFRSF14, ADIPOR2, EFNB2, B4GALNT1, CCNI, NEUROD2, IHH |
